# Wuling powder ameliorates diarrhea-predominant irritable bowel syndrome in mice by modulating gut mucosal microbiota and alleviating intestinal inflammation

**DOI:** 10.3389/fcimb.2025.1652186

**Published:** 2025-08-14

**Authors:** Qianghong Tian, Donglin Yu, Junxi Shen, Leyao Fang, Ying Cai

**Affiliations:** School of Traditional Chinese Medicine, Hunan University of Chinese Medicine, Changsha, Hunan, China

**Keywords:** Wuling powder, diarrhea predominant-irritable bowel syndrome, intestinal mucosal microbiota, 16S rRNA, TCM, inflammation

## Abstract

**Objectives:**

Wuling powder has been widely used for the clinical treatment of IBS-D, but the microecological mechanism has not been found. This study aimed to investigate the regulatory effect of Wuling powder on intestinal mucosal microbiota in mice with diarrhea predominant-irritable bowel syndrome (IBS-D) by 16S rRNA high-throughput sequencing.

**Methods:**

Mice were randomly divided into normal control group (Mc), model control group (Mm), and treatment group (Mt). The IBS-D model was induced via *Folium sennae* gavage and acute restraint stress, and treatment was conducted with Wuling powder. Serum levels of tumor necrosis factor-α (TNF-α), interleukin-6 (IL-6), and milk fat globule EGF factor 8 (MFGE8) were quantified via enzyme-linked immunosorbent assay (ELISA). Intestinal mucosal DNA was extracted for 16S rRNA gene high-throughput sequencing to analyze microbial community alterations. We analyzed the characteristics of the intestinal mucosal microbiota and explored the potential link between the intestinal mucosal microbiota and the inflammatory response.

**Results:**

Compared with the Mc group, the Mm group presented markedly elevated serum TNF-α and IL-6 concentrations (*p*<0.001; *p*<0.001) and significantly decreased MFGE8 levels (*p*<0.01). In contrast, the Mt group presented significant reductions in TNF-α and IL-6 (*p*<0.05; *p*<0.05) and a pronounced increase in MFGE8 (*p*<0.01) relative to those in the Mm group. After treatment with Wuling powder, the abundance and diversity of the intestinal mucosal microbiota were restored. The characteristic genus *Sporosarcina* was significantly positively correlated with MFGE8, and *Paludibaculum* was significantly negatively correlated with TNF-α and IL-6.

**Conclusion:**

Wuling powder may inhibit the occurrence of an inflammatory response by reducing the TNF-α and IL-6 levels and increasing the MFGE8 level and may achieve the effect of treating IBS-D by regulating the composition, structure, and function of the intestinal mucosal microbiota, which provides new ideas for the clinical prevention and treatment of IBS-D via Wuling powder.

## 1 Introduction

Irritable bowel syndrome (IBS) is a functional gastrointestinal disorder characterized by chronic abdominal pain (≥6 months) with at least 3 months of persistent symptoms, accompanied by altered bowel habits or abnormal defecation (Grayson, 2016). IBS are clinically classified into four subtypes: diarrhea-predominant IBS (IBS-D), constipation-predominant IBS, mixed IBS, and unclassified IBS, of which IBS-D is the most common subtype in the Rome IV criteria for IBS (Grayson, 2016; Mearin et al., 2016). Epidemiological data indicate a 2.3-15.8% prevalence of IBS in China, with significant female predominance and higher incidence of IBS-D (Yin et al., 2022). The pathophysiology of IBS-D involves multifactorial mechanisms including intestinal dysmotility, visceral hypersensitivity, brain-gut axis dysregulation, gut microbial imbalance, and impaired mucosal barrier integrity (Bellini et al., 2014; Saha, 2014). In TCM, chronic use of *Folium sennae*, a cold-natured purgative, may induce spleen qi deficiency through prolonged cold-induced impairment. We developed an IBS-D model by combining morning oral administration of *Folium sennae* with afternoon restraint-tail clamping stress. This dual intervention simulates hepatic qi stagnation via psychosocial stress-induced anxiety behaviors, subsequently triggering liver-spleen disharmony to exacerbate splenic insufficiency (Liu et al., 2020; Peng and Xu, 2021).

The gut microbiota, a dynamic community of roughly 4×10^^13^ bacterial cells, is crucial for host metabolism and immune function. Notably, the Bacteroidetes and Firmicutes phyla dominate this ecosystem, comprising over 70% of the microbial population, with their abundance and diversity strongly linked to long-term dietary habits (Canakis et al., 2020). Under physiological conditions, gut microbiota maintains host-environment equilibrium. Dysbiosis disrupts metabolic/immune functions, triggering IBS-D via barrier disruption, immune activation, and visceral hypersensitivity (Yuan et al., 2023; Chen et al., 2024).

IBS-D-associated intestinal mucosal immune dysfunction disrupts both motility and barrier function, with TNF-α, IL-6, and MFGE8 serving as key mediators linking inflammatory signaling, apoptosis regulation, and barrier repair pathways. Studies indicate that (Qu et al., 2023) immune dysregulation in the intestinal mucosa of IBS-D patients involves heightened inflammatory cell infiltration and elevated proinflammatory cytokines, including TNF-α and IL-6. These mediators modulate gut motility via neuroendocrine pathways, contributing to abdominal pain, bloating, and altered bowel habits. Research indicates that (Kasti et al., 2025) TNF-α in IBS-D patients exacerbates intestinal inflammation and immune dysfunction, worsening symptoms and increasing risks of mucosal damage and disease recurrence. MFG-E8 alleviates inflammation by inhibiting NF-κB, regulating NLRP3, polarizing anti-inflammatory macrophages, and reducing DAMPs, thereby decreasing TNF-α and IL-6 (Hua et al., 2022). Though mechanisms require clarification, MFG-E8 targeting may offer new IBS-D treatment.

IBS-D has complex pathophysiology. Current therapies (e.g., antidiarrheals, antispasmodics, gut-brain modulators) only alleviate symptoms rather than addressing the root cause, often with high costs and poor adherence (Galica et al., 2022). Wuling powder is derived from the “Treatise on Typhoid Fever”. It consists of *Poria cocos* (Schw.)Wolf, *Polyporus umbellatus*(Pers.)Fries, *Alisma*(Sam.)Juzep., *Atractylodes macrocephala* Koidz. and *Cinnamomi Ramulus*. Clinically used for water-dampness retention presenting as diarrhea, edema, and dysuria. Clinical studies demonstrate that modified Wuling power effectively treats IBS-D by alleviating symptoms, improving life quality, and reducing relapse (Lin and Guo, 2020). Its bioactive components (e.g., *Poria cocos* (Schw.) Wolf, *Atractylodes macrocephala Koidz* polysaccharide) exert anti-inflammatory, antioxidant, and microbiota-modulating effects, supporting its therapeutic use (Ding et al., 2024; Zhang et al., 2025). The Wuling powder decoction was selected for its superior efficacy and faster action than pill forms. TCM for IBS-D demonstrates unique advantages through syndrome differentiation and multi-target mechanisms, modulating gut microbiota to restore intestinal homeostasis, enhance barrier function, and reduce inflammation, thereby alleviating symptoms (Jun et al., 2022). Numerous experimental studies have demonstrated that TCM exhibits significant therapeutic efficacy in the treatment of IBS-D (Zhao et al., 2024; Wang et al., 2025). Clinical studies (Gao, 2019; Xu, 2021) confirm Wuling power’s efficacy in alleviating gastrointestinal disorders, particularly IBS-D. A stable microbial environment is critical for host health (Liu et al., 2021; Quaglio et al., 2022). Clinical observations and animal experimental studies confirm that IBS-D models alter gut microbiota composition, structure and increase intestinal permeability (Tanaka et al., 2022; Wu H. et al., 2022). Furthermore, Hong et al. (2020) found fungal dysbiosis and disrupted bacterial-fungal interactions in IBS-D patients, revealing complex microbial imbalances.

Although clinical studies confirm the efficacy of Wuling power in treating IBS-D, its mechanisms in modulating gut microbiota and inflammatory responses remain unclear. Recent advances in 16S rRNA sequencing have enabled microbiome research on herbal compounds (Li et al., 2022; Yi et al., 2023), yet no study has examined induced microbial shifts in IBS-D. Here, we established an IBS-D mice model, analyzed mucosal microbiota changes via 16S rRNA sequencing, and explored their correlation with intestinal inflammation. This study investigates the microecological mechanisms underlying the therapeutic effects of Wuling powder in IBS-D, aiming to establish a theoretical foundation for its clinical application in IBS-D treatment.

## 2 Materials and methods

### 2.1 Experimental animals, grouping and feeding conditions

To exclude the effects of sex on the intestinal microbiota of the mice (Wu Y. et al., 2022), eighteen specific-pathogen-free Kunming mice (4-week-old males) were purchased from Hunan Slaccas Jingda Laboratory Animal Company (Hunan, China) with the license number SCXK (Xiang) 2019-0004. These animals were housed in sterile individual cages lined with clean bedding within the barrier environment of the Experimental Animal Center at Hunan University of Chinese Medicine (License No.: SYXK (Xiang) 2019-0009). Cages were changed weekly, with environmental conditions maintained at a temperature of 23–25°C, relative humidity of 50–70%, and a 12-hour light/dark cycle. Throughout the experimental period, the mice had ad libitum access to autoclaved water and standard rodent chow provided by the animal center. The experimental feed was provided by the Laboratory Animal Center of Hunan University of Chinese Medicine and manufactured by Beijing Huafu Biotechnology Co., Ltd. The formulation comprised nutritionally balanced components, including corn, soybean meal, wheat middlings, fish meal, soybean oil, limestone, sodium phosphate dibasic, multivitamin premix, and multiple mineral elements, among others. The feed was certified under the Beijing Feed Production License (2024)06076, meeting strict hygienic standards with a guaranteed absence of contaminants. **Table 1** shows the nutritional guarantee values expressed per kilogram of complete feed, including proximate analysis and micronutrient specifications. All animal experiments and procedures complied with the standards of the Animal Ethics and Welfare Committee of Hunan University of Chinese Medicine with permission number LL2021111004.

**Table 1:** Nutritional composition of the experimental diet (g/kg diet).

Nutrient	Weight (g)	Nutrient	Weight (g)
Crude Protein	≥180	Calcium	10-18
Crude Fat	≥40	Phosphorus	6-12
Moisture	≤100	Lysine	≥8.2
Crude Ash	≤80	Methionine + Cysteine	≥5.3
Crude Fiber	≤50	Vitamin E	≥0.04

### 2.2 Medicine and preparation process

According to the Chinese Pharmacopoeia 2020 edition, Wuling power is composed of the following herbal components: *Poria cocos* (Schw.) Wolf (produced by Hunan Haosheng Chinese Herbal Pieces Co., Ltd., Changsha, China, batch number: 240703), *Polyporus umbellatus* (Pers.) Fries (produced by Hunan Rongkang Chinese Herbal Pieces Co., Ltd., Changsha, China, batch number: 240401), *Alisma* (Sam.) Juzep. (produced by Anhui Shenghaitang Chinese Herbal Pieces Co., Ltd., Bozhou, China, batch number: 2024070345), *stir-fried Atractylodes macrocephala* Koidz. (produced by Hunan Junhao Chinese Herbal Science and Trade Co., Ltd., Changsha, China, batch number: 240702), and *Cinnamomi Ramulus* (produced by Hunan Rongkang Chinese Herbal Pieces Co., Ltd., Changsha, China, batch number: 240401). Additionally, *Folium sennae* (produced by Bozhou Huqiao Pharmaceutical Co., Ltd., Bozhou, China; batch number: 2311300022) was included in the study. All the herbal materials used were procured from the First Affiliated Hospital of Hunan University of Chinese Medicine. The detailed formulation and compositional ratios are provided in **Table 2**. The listed quantities of the Wuling power components correspond to one standard dose of the decoction.

**Table 2:** Composition of the Wuling powder.

Chinese name	Latin name	Place	Part used	Amount (g)
Fuling	*Poria cocos* (Schw.) Wolf	Yunnan	Nucleus	15
Zhuling	*Polyporus umbellatus* (Pers.) Fries	Shanxi	Nucleus	15
Zexie	*Alisma orientale* (Sam.) Juzep.	Sichuan	Root	15
Baizhu	*Atractylodes macrocephala* Koidz.	Zhejiang	Rhizome	10
Guizhi	*Cinnamomi Ramulus*	Guangxi	tender branch	6

IBS, irritable bowel syndrome; IBS-D, diarrhea predominant-irritable bowel syndrome; TCM, traditional Chinese medicine; TNF-α, tumor necrosis factor-α; IL-6, interleukin-6; MFGE8, milk fat granule EGF factor 8; OD, optical density; NF-κB, nuclear factor kappa-B; PCoA, principal coordinate analysis; NMDS, nonmetric multidimensional scaling; ANOSIM, analysis of similarities; LEfSe, linear discriminant analysis effect size; ROC, receiver operating characteristic; AUC, area under the curve; KEGG, Kyoto Encyclopedia of Genes and Genomes; OTU, operational taxonomic unit; RDA, redundancy analysis; ANOVA, one-way analysis of variance; LSD, least significant difference; ANOSIM, analysis of similarities; FMT, fecal microbiota transplantation; CD, Crohn’s disease.

Preparation of *Folium sennae* decoction: According to previous methods (Hu et al., 2018), we steeped 100 g of *Folium sennae* with 1L of boiling water for 10 min (the whole leaf was soaked without removing the petiole). The liquid was subsequently filtered through gauze, evaporated to 1 g/mL in a 70°C rotary evaporator, and finally stored at 4°C.

Preparation of the Wuling powder decoction: The medicine was weighed according to the above ratio, and cold water was added to soak the surface of the medicine for 30 min. The medicine was decocted with a large flame until it boiled and then with a soft flame. The decoction time was 20–30 min. The medicine was successively decocted two times and then filtered through gauze. The liquid medicine was decocted two times to make the Wuling powder decoction, which was evaporated to 0.34 g/mL in a 75°C water bath. The mixture was finally stored at 4°C.

### 2.3 Experimental animal grouping, modeling, and treatment

**Figure 1:**
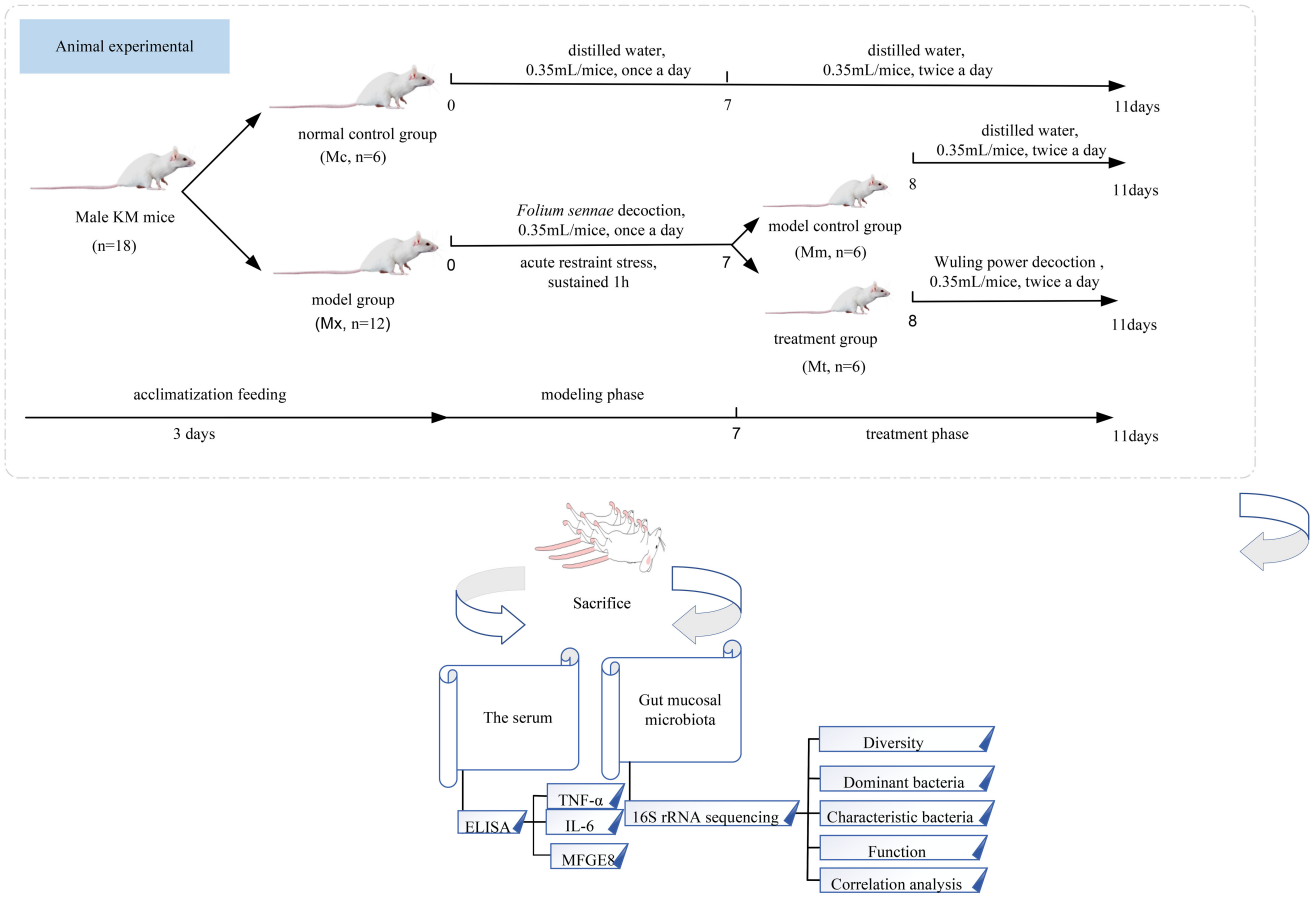
Experimental flow chart. Eighteen KM mice were randomly assigned to the Mc group and the Mx group. Mice in the Mx group underwent modeling for 7 days through Folium Sennae gavage and acute restraint stress. Subsequently, the Mx group mice were further randomly divided into the Mm group and the Mt group. Mice in the Mt group received treatment with Wuling powder for 4 days. Finally, samples were collected from all groups. Serum inflammatory factors (TNF-α, IL-6, MFGE8) were analyzed using ELISA, and the intestinal mucosal microbiota was assessed via 16S rRNA sequencing. Mc, normal control group; Mm, model control group; Mt, treatment group.

The experimental procedure involving animals is schematically illustrated in **Figure 1**. Eighteen mice were allowed free access to water and maintained on a standard diet for 3 days prior to randomization. These animals were subsequently randomly allocated to the normal control group (Mc, n=6) and model group (Mx, n=12). In reference (Liu et al., 2020; Peng and Xu, 2021), the IBS-D model was constructed by combining *Folium sennae* with the restraint tail method. During the experimental period (Li et al., 2025), the specific procedures were as follows: At 9:00 AM daily, the Mx group received intragastric administration of *Folium sennae* decoction (0.35 mL per mouse each time) to establish spleen deficiency-diarrhea syndrome, whereas the Mc group received equivalent volumes of distilled water. At 3:00 PM, the Mx group mice were subjected to 1 hour of restraint stress by immobilizing their limbs in centrifuge tubes and clamping the distal third of their tails with long-tail clips, whereas the Mc group remained untreated in the afternoon. Successful model establishment, indicated by marked irritability and diarrhea in mice, was achieved following 7 days of protocol implementation across all groups. Following successful model establishment, the 12 Mx mice were randomly divided into the model control group (Mm, n=6) and the treatment group (Mt, n=6). The Mt group received Wuling power at a dosage of 7.93 g/(kg·d) decoction via gavage (0.35 mL/mice) for 4 days, twice a day. The Mc and Mm groups received equivalent volumes and frequencies of distilled water during this intervention period.

### 2.4 General behavioral observation

During the modeling and administration periods, we observed the behavioral status of the mice in all the groups and recorded emotional changes, activity status, coat quality, fecal characteristics, and perianal cleanliness.

### 2.5 Determination of tumor necrosis factor-α, interleukin-6, and milk fat globule EGF factor 8 in serum

Under aseptic conditions, the mice were euthanized by cervical dislocation, followed immediately by blood collection via retro-orbital bleeding. Whole blood samples from each group were collected in 1.5 mL microcentrifuge tube and allowed to stand for phase separation. After overnight incubation at 4°C, the samples were subjected to low-temperature high-speed centrifugation (4°C, 3000 r/min) for 15 minutes. Supernatants were collected via micropipette and transferred into sterile centrifuge tubes for subsequent analysis. Strictly adhering to the ELISA kit manufacturer’s protocols, all kits were equilibrated at room temperature for 30 minutes prior to use. The procedure encompassed plate layout configuration, sample loading, enzyme conjugate addition, incubation, plate washing, color development, reaction termination, and instrumental detection. Standard curves were generated by plotting the optical density (OD) values (y-axis) against the corresponding standard concentrations (x-axis), followed by linear regression analysis. The resultant curve equations were subsequently employed to calculate the concentration levels of TNF-α, IL-6, and MFGE8 in serum samples. The specifications of the commercial kits utilized were as follows: Mouse TNF-α ELISA Kit (batch number: A115357; Shanghai Fusheng Industrial Co., Ltd., China), Mouse IL-6 ELISA Kit (batch number: A105582; Shanghai Fusheng Industrial Co., Ltd., China), and Mouse MFGE8 ELISA Kit (batch number: A106326; Shanghai Fusheng Industrial Co., Ltd., China). ELISA is a highly sensitive immunoassay that quantifies or detects target molecules (e.g., proteins, hormones, pathogens) via antigen-antibody binding and enzyme-substrate signal amplification. Due to its high specificity and versatility, it is widely used in biomedical research, clinical diagnostics, and drug development.

### 2.6 Total DNA preparation and 16S rRNA high-throughput sequencing

Following euthanasia via cervical dislocation under aseptic conditions, small intestine tissues were excised from the mice. The luminal levels were gently extruded, and the intestinal segments were systematically dissected. Intestinal mucosal samples were collected from sterile cryovials, immediately snap-frozen in liquid nitrogen, and maintained at -80°C until 16S rRNA high-throughput sequencing analysis (Guo et al., 2025; Shen et al., 2025a). All the experimental procedures were conducted by Beijing Biomarker Technologies Co., Ltd. (Beijing, China). Total genomic DNA was extracted from intestinal mucosa samples via the MN NucleoSpin 96 Soil DNA Extraction Kit according to the manufacturer’s protocols. The hypervariable V3–V4 regions of the bacterial 16S rRNA genes were amplified with the universal primers 338F (5’-ACTCCTACGGGAGGCAGCA-3’) and 806R (5’-GGACTACHVGGGTWTCTAAT-3’) through two-stage PCR amplification (regional PCR followed by Solexa PCR). The amplification success was verified by 1.8% agarose gel electrophoresis (120 V, 40 min), with band intensity quantification performed via ImageJ software. Equimolar concentrations of PCR products were pooled and purified via the OMEGA DNA Clean-Up Kit. The target fragments were excised from the 1.8% agarose gels and recovered with the Monarch DNA Gel Extraction Kit. Library construction and sequencing were performed by Beijing Biomarker Technologies Co., Ltd., on the Illumina NovaSeq platform. The raw sequencing data of the intestinal mucosal microbiota have been deposited in the NCBI Sequence Read Archive (National Center for Biotechnology Information, https://www.ncbi.nlm.nih.gov/) under accession number PRJNA951492.

### 2.7 Bioinformatics

#### 2.7.1 Sequencing data quality assessment

The qualified libraries were sequenced via the Illumina NovaSeq 6000 platform to generate raw reads. Initially, the raw sequencing reads were subjected to quality filtering via Trimmomatic v0.33. The primer sequences were subsequently identified and removed via Cutadapt 1.9.1 software to obtain primer-free clean reads. The clean reads from each sample were then processed for overlapping assembly via Usearch v10, followed by length-based filtration of the assembled sequences according to region-specific amplicon length ranges. Chimeric sequences were detected and eliminated via UCHIME v4.2 to yield high-quality effective reads. Data quality assessment was performed through three analytical metrics: rarefaction curves for evaluating sequencing depth, Shannon–Wiener curves for determining sampling sufficiency, and coverage indices for verifying sample homogeneity. These analyses collectively ensured the reliability of the sequencing data for subsequent bioinformatic investigations.

#### 2.7.2 Microbial structure analysis

Alpha diversity refers to the species diversity within a specific region or ecosystem. Community richness and diversity were assessed through the calculation of the ACE index, Chao1 index, Shannon index, and Simpson index. The QIIME2 platform (https://qiime2.org/) was used for alpha diversity analysis. Beta diversity describes compositional dissimilarities in species communities across distinct habitats. Ordination analyses, including principal coordinate analysis (PCoA) and nonmetric multidimensional scaling (NMDS), were performed to visualize sample relationships by projecting multidimensional data into lower-dimensional spaces while maximizing intersample distances and preserving relational information in planar scatterplots. Graphical representations were generated via OmicStudio tools (https://www.omicstudio.cn/tool). Furthermore, analysis of similarities (ANOSIM) was conducted to statistically determine whether significant differences existed in community composition between distinct groups.

#### 2.7.3 Microbial composition analysis

Sequence clustering was performed via the USEARCH platform (version 10.0) with a 97% similarity threshold, followed by OTU filtering via a default threshold of 0.005% of the total sequenced reads. Bar charts, a conventional visualization method for characterizing multisample taxonomic compositions, were generated through the GenesCloud bioinformatics platform (https://www.genescloud.cn/chart/ChartOverview) to illustrate the microbial community distribution patterns at both the phylum and genus levels across the experimental groups.

#### 2.7.4 Characteristic bacteria analysis

Random forest classifier and linear discriminant analysis effect size (LEfSe) analyses are widely employed for screening biologically significant biomarkers associated with group stratification (Shen et al., 2025c). The random forest model finds the key species that can distinguish the differences between two groups by mining the nonlinear interdependence between variables. Moreover, LEfSe identifies the species that significantly impact sample division among multiple groups by performing linear discriminant analysis on samples according to different groups. To evaluate the diagnostic efficacy of the feature bacterial genera selected by random forest, receiver operating characteristic (ROC) curves of characteristic bacteria were constructed, and the area under the curve (AUC) was calculated. The AUC is generally between 0 and 1. The closer the AUC is to 1, the greater the number of microbiota with diagnostic efficacy in both groups.

### 2.8 Functional prediction analysis

PICRUSt2 is a computational method that uses marker gene data and a genomic reference database to predict the functional composition of environmental microorganisms (Fang et al., 2025). The Kyoto Encyclopedia of Genes and Genomes (KEGG) was classified via PICRUSt2. Metabolic pathway visualization was subsequently performed via the Paisenoruo Cloud Platform (https://www.genescloud.cn/chart/ChartOverview) for comprehensive graphical representation.

### 2.9 Correlation analysis

We calculated Spearman’s correlation coefficients between intestinal mucosal characteristic genera and inflammation-related factors. Subsequently, redundancy analysis (RDA) and correlation scatter plots were used to investigate the interactions between characteristic genera and inflammation-related factors (Di et al., 2025). All correlation analyses and visualizations were conducted via OmicStudio tools (https://www.omicstudio.cn/tool), a validated bioinformatics platform for microbial-omics integration.

### 2.10 Statistical analysis

Excel and SPSS 25.0 software were used to process and analyze the data. GraphPad Prism 8.0 software was used to plot the images. SPSS 25.0 software was used for the statistical analysis, and the data obtained from each group are expressed as the means ± standard deviations (means ± SD). If the data conformed to a normal distribution and homogeneity of variance, the independent sample test was used for comparative analysis between two groups; one-way ANOVA was used for comparative analysis between multiple groups; and the least significant difference (LSD) method was used for two-way comparisons between groups. Otherwise, the Mann–Whitney U test was used for comparisons between two groups, and the Kruskal–Wallis H test was used for comparisons between multiple groups. The test level was set at α=0.05. When *p*<0.05, the results were significantly different; when *p*<0.01, the results were extremely significantly different (Yu et al., 2024; Shen et al., 2025b).

## 3 Results

### 3.1 Effects of Wuling power on general behavior in a murine model of IBS-D

**Figure 2:**
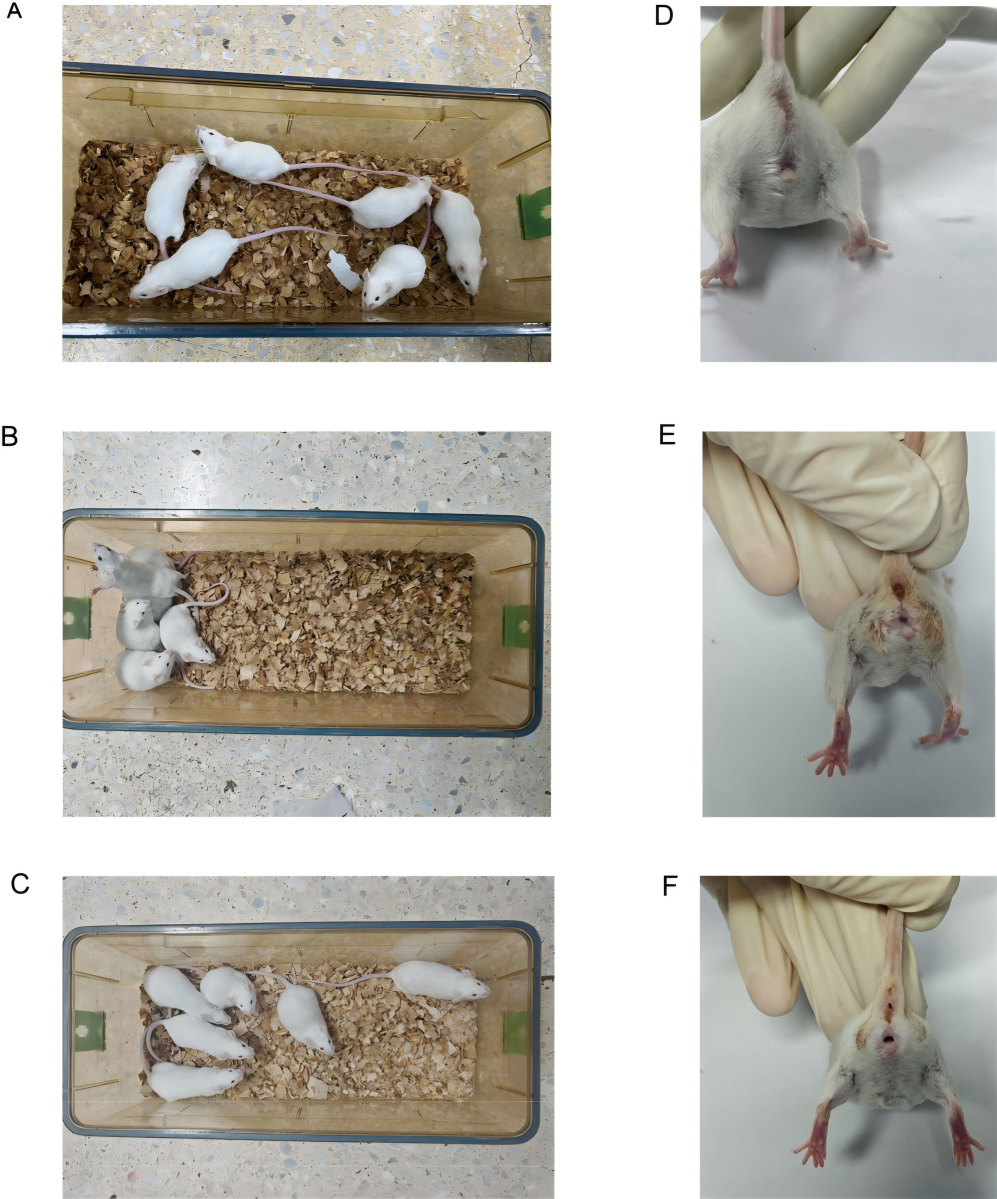
Effects of Wuling powder on the general behavior of IBS-D mice. **(A)** Behavioral and activity states of Mc group mice. **(B)** Behavioral and activity states of the mice in the Mm group. **(C)** Behavioral and activity states of Mt group mice. **(D)** Perianal condition of Mc group mice. **(E)** Perianal condition of the mice in the MM group. **(F)** Perianal condition of Mt group mice.

During the experimental period, the mice in the Mc group maintained a normal mental status and spontaneous activity, with dry bedding, smooth and glossy fur, well-formed stools of appropriate softness, and clean perianal regions (**Figures 2A, D**). In contrast, the mice in the Mm group developed progressively loose and watery stools during model induction, accompanied by increased irritability, piloerection, fighting behavior with audible vocalizations, increased resistance to gavage, reduced spontaneous locomotion, and huddling tendencies. These animals exhibited damp bedding, disheveled fur, viscous and poorly formed stools, and fecal contamination around the anal area (**Figures 2B, E**). Following Wuling power administration, the Mt group demonstrated gradual improvement in mental state, increased locomotor activity, and reduced huddling behavior as the treatment duration progressed (**Figures 2C, F**). However, the therapeutic outcomes remained statistically inferior to those of normal controls. These observations suggest that Wuling power has moderate therapeutic efficacy in IBS-D model mice, although complete restoration of physiological and behavioral parameters was not achieved.

### 3.2 Effects of Wuling power on serum levels of IL-6, TNF-α, and MFGE8 in IBS-D mice

**Figure 3:**
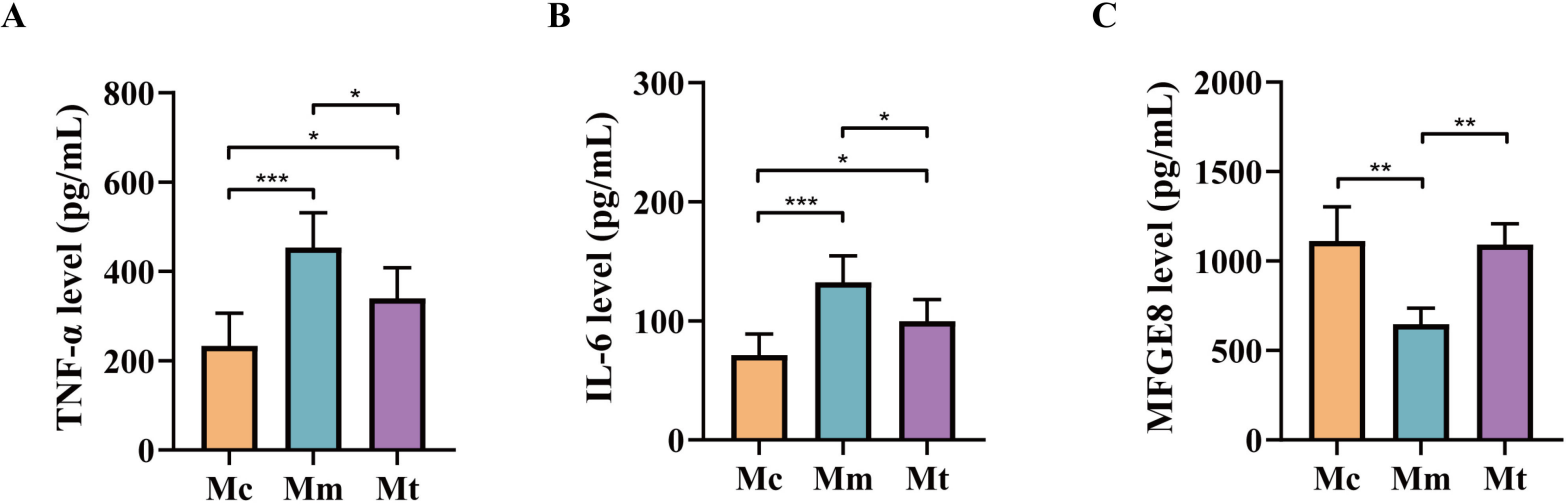
Effects of Wuling powder on relevant environmental factors in IBS-D mice. **(A)** TNF-α level; **(B)** IL-6 level; **(C)** MFGE8 level. Mc, normal control group; Mm, model control group; Mt, treatment group. *p<0.05; **p<0.01; ***p<0.001.

Pro-inflammatory factors (e.g., IL-6, TNF-α) and anti-inflammatory factors (e.g., MFGE8) exhibit antagonistic yet coordinated actions, collectively regulating inflammation initiation, progression, and resolution (Tao et al., 2022). As shown in **Figures 3A–C**, the levels of TNF-αand IL-6 in the serum were significantly greater (*p*<0.001; *p*<0.001) in the Mm group than in the Mc group, whereas the MFGE8 level was significantly lower (*p*<0.01). Compared with those in the Mm group, the levels of TNF-αand IL-6 in the Mt group were significantly lower (*p*<0.05; *p*<0.05), whereas the MFGE8 level was significantly greater (*p*<0.01). These results indicate that the model induced an intestinal inflammatory response in mice and that treatment with Wuling powder effectively alleviated intestinal inflammation.

### 3.3 Analysis of the effects of Wuling powder on the intestinal mucosal microbiota in IBS-D mice

#### 3.3.1 Quality assessment of sequencing data

**Figure 4:**
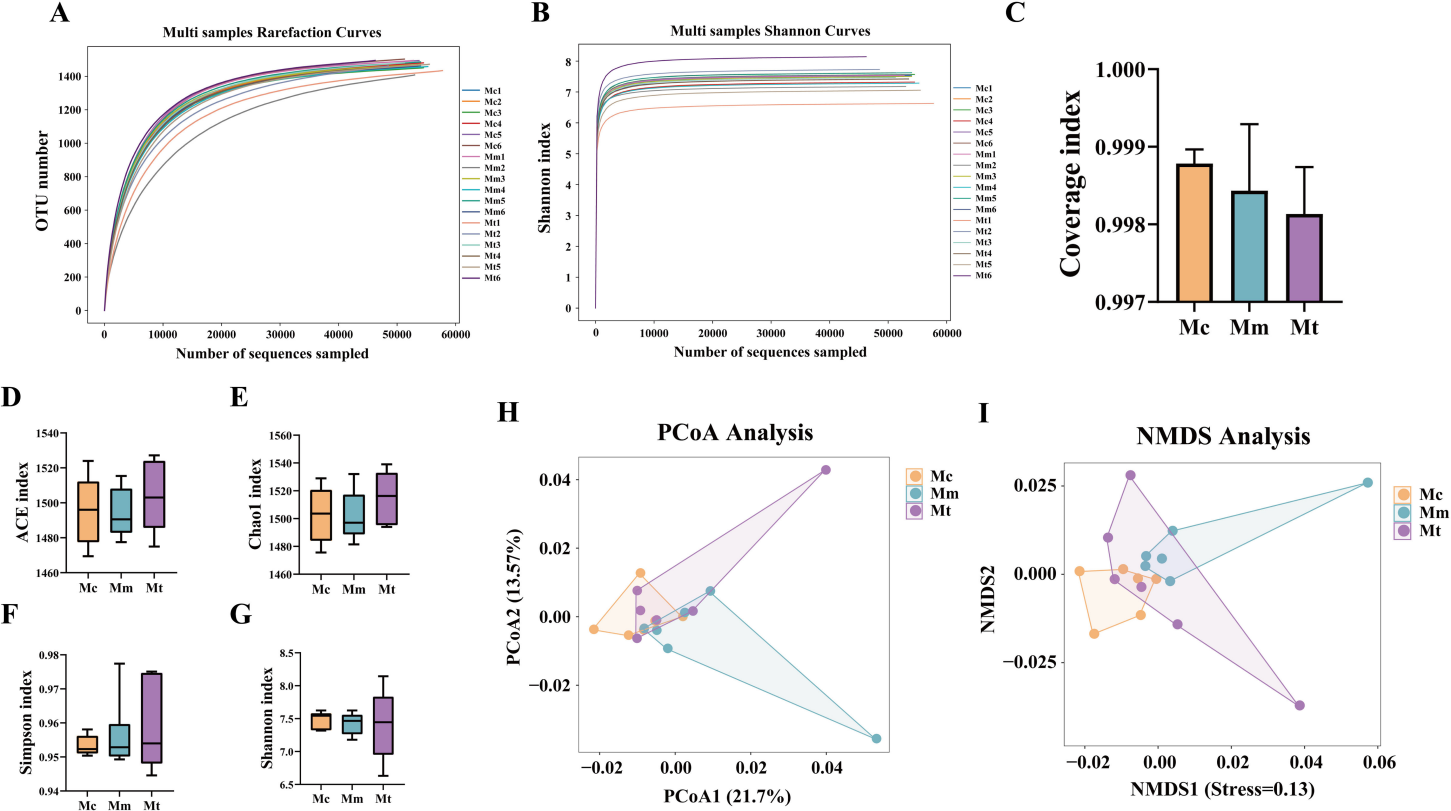
Effects of Wuling powder on alpha and beta diversity in IBS-D mice. **(A)** Rarefaction curve; **(B)** Shannon–Wiener curve; **(C)** Coverage index; **(D)** ACE index; **(E)** Chao1 index; **(F)** Simpson index; **(G)** Shannon index; **(H)** PCoA analysis; **(I)** NMDS analysis. Mc, normal control group; Mm, model control group; Mt, treatment group.

As shown in **Figures 4A, B**, with increasing number of sequencing reads, the dilution curves and Shannon–Wiener curves in this study gradually flattened, indicating that further increases in sequencing depth would yield only a limited number of additional microbial species. This suggests that the sequencing depth for the samples was sufficient and reasonable, covering the majority of the biological species. The species richness of the detected samples was adequate to meet the requirements of subsequent research. Increasing the sequencing depth further would not significantly alter the current detection results. In this study, the coverage index was introduced to evaluate the coverage of samples within groups. The results revealed that the coverage indices of samples within the same group were all above 0.997, indicating good coverage within the group and no outliers with excessively large deviations. This suggests that the sequences in the samples were almost fully detected, confirming that the samples met the requirements of the experimental design (**Figure 4C**).

#### 3.3.2 Wuling powder affects the diversity, richness, and microbial structure of the intestinal mucosal microbiota in IBS-D mice

As shown in **Figures 4D–G**, the ACE and Chao1 indices were ranked from low to high as follows: Mm group<Mc group<Mt group (*p*>0.05). The Simpson index was ranked from low to high as the Mc group<Mm group<Mt group (*p*>0.05). The Shannon index was ranked from low to high as Mt group <Mm group<Mc group (*p*>0.05). These results indicated that alpha diversity was reestablished following modeling and treatment. Beta diversity, which describes the differences in species composition between communities in different habitats, was analyzed to compare variations among different samples. As shown in **Figures 4H, I**, significant differences were observed in the microbial community composition among the groups (*p*=0.034), as confirmed by the ANOSIM test. Clusters closer to each other on the coordinate axes presented greater similarity in community composition along the corresponding dimensions. PCoA revealed that PCoA1 contributed 21.7%, and PCoA2 contributed 13.57% of the variation (**Figure 4H**). Notably, the Mc group samples exhibited minimal overlap with the Mm group samples, suggesting that modeling induced certain alterations in the gut mucosal microbiota structure of the mice. The Mt group samples showed limited overlap with the Mm group samples and were closer in distance to the Mc group, indicating lower similarity between the Mt and Mm groups and greater similarity between the Mt and Mc groups. These findings suggest that the administration of Wuling powder may have a restorative effect on the structure of the gut mucosal microbiota. The NMDS analysis (**Figure 4I**) corroborated the PCoA findings. Collectively, these results demonstrate that Wuling powder influences the diversity, richness, and microbial structure of the gut mucosal microbiota in IBS-D model mice.

#### 3.3.3 Wuling powder affects the composition of the intestinal mucosal microbiota in IBS-D mice

**Figure 5:**
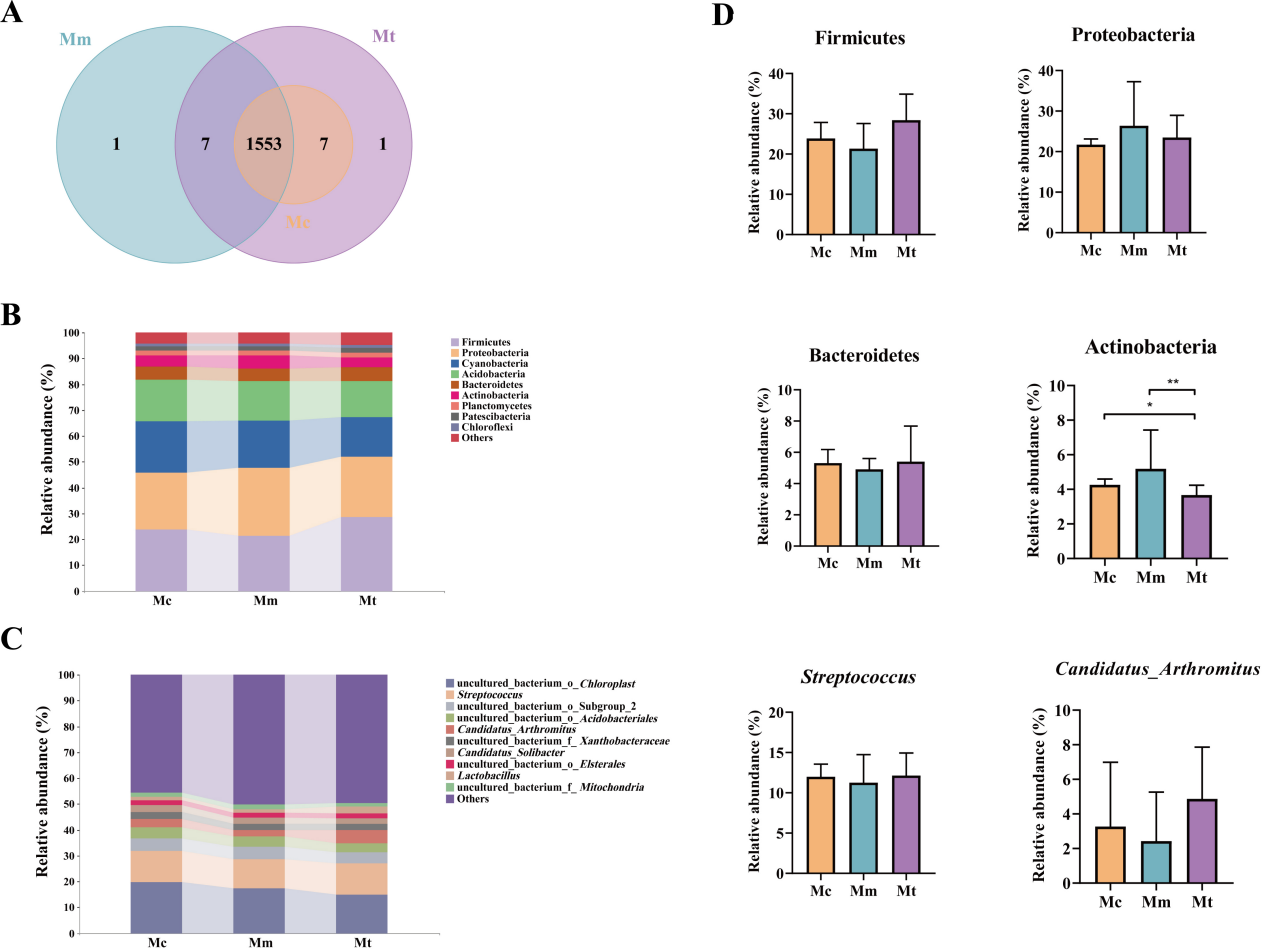
Effects of Wuling powder on the intestinal mucosal microbiota structure in IBS-D mice. **(A)** OTU number; **(B)** Histogram of relative abundance at the phylum level; **(C)** Histogram of relative abundance at the genus level; **(D)** Phyla and genera with changing trends. Mc, normal control group; Mm, model control group; Mt, treatment group. *p<0.05; **p<0.01.

Sequence analysis was performed via USEARCH (version 10.0) with a similarity threshold of 97% for clustering. The default threshold for filtering OTUs was set at 0.005% of the total number of sequences. The Mc group identified 1560 OTUs, with no unique OTUs. The Mm group identified 1561 OTUs, including 1 unique OTU, whereas the Mt group identified 1568 OTUs, with 8 unique OTUs. A total of 1553 OTUs were shared among the three groups (**Figure 5A**). We selected the top 10 bacterial phyla and genera with relative abundances exceeding 1% and presented their distributions in bar charts (**Figures 5B, C**). As shown in **Figure 5B**, Firmicutes, Proteobacteria, Cyanobacteria, Acidobacteria, Bacteroidetes, and Actinobacteria were the dominant phyla across all three groups. Statistical analysis of these dominant phyla revealed the following trends (**Figure 5D**). Compared with those in the Mc group, Firmicutes and Bacteroidetes in the Mm group tended to decrease (23.90 vs. 21.35%, *p*>0.05; 5.31 vs. 4.91%, *p*>0.05), whereas Proteobacteria and Actinobacteria tended to increase (21.73 vs. 26.40%, *p*>0.05; 4.26 vs. 5.19%, *p*>0.05). Compared with those in the Mm group, Firmicutes and Bacteroidetes were increased in the Mt group (21.35 vs. 28.43%, *p*>0.05; 4.91 vs. 5.40%, *p*>0.05), whereas Proteobacteria and Actinobacteria were decreased (26.40 vs. 23.48%, *p*>0.05; 5.19 vs. 3.67%, *p*=0.029). **Figure 5C** shows the relative abundances of the dominant genera in the gut mucosal microbiota of the mice. The predominant genera included *uncultured_bacterium_o_Chloroplast*, *Streptococcus*, *uncultured_bacterium_o_Subgroup_2*, *uncultured_bacterium_o_Acidobacteriales*, and *Candidatus_Arthromitus*. As shown in **Figure 5D**, *Streptococcus* and *Candidatus_Arthromitus* were lower in the Mm group than in the Mc group (11.99 vs. 11.26%, *p*>0.05; 3.27 vs. 2.43%, *p*>0.05). Compared with those in the Mm group, Streptococcus and Candidatus Arthromitus were increased in the Mt group (11.26 vs. 12.14%, *p*>0.05; 2.43 vs. 4.87%, *p*>0.05). In conclusion, the administration of Wuling powder induced changes in the composition of the dominant mucosal microbiota at both the phylum and genus levels in IBS-D mice.

#### 3.3.4 Analysis of the effects of Wuling powder on the characteristic genera of the intestinal mucosal microbiota in IBS-D mice

**Figure 6:**
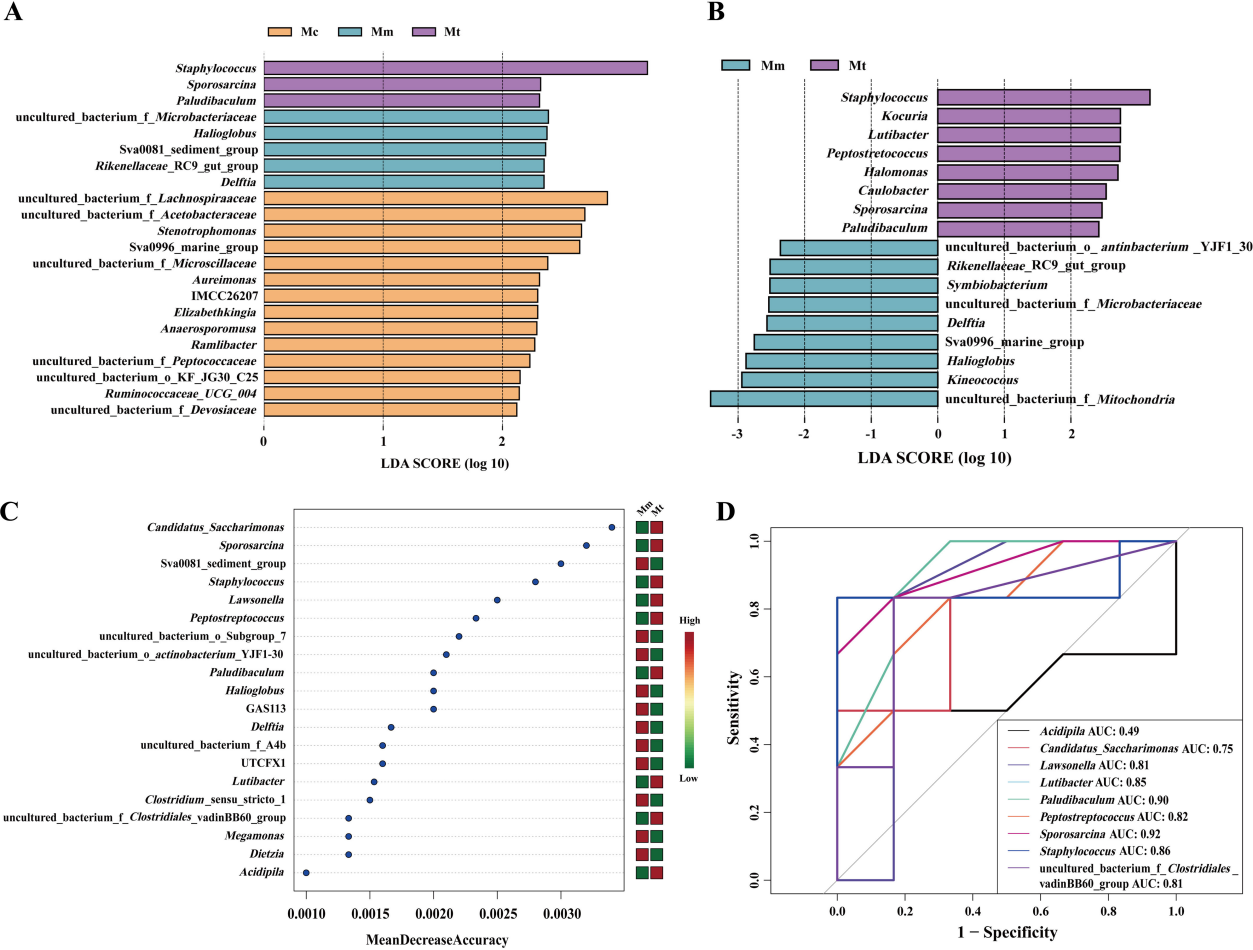
Effects of Wuling powder on the characteristics of the intestinal mucosal microbiota in IBS-D mice. **(A)** LEfSe analysis of the genus level among the Mc, Mm, and Mt groups; **(B)** LEfSe analysis of the genus level between the Mm and Mt groups; **(C)** Random forest analysis of the genus level in the Mt group; **(D)** ROC curve analysis of the genus level in the Mt group. Mc, normal control group; Mm, model control group; Mt, treatment group.

LEfSe analysis and random forest analysis are commonly used to identify key biomarkers that significantly influence group differentiation. To further validate the regulatory effect of Wuling powder on the gut mucosal microbiota of IBS-D mice, LEfSe analysis was employed, with a linear discriminant analysis (LDA) score>2 as the selection criterion. By performing linear discriminant analysis on samples under different grouping conditions, we identified microbial species that significantly contributed to the differentiation among multiple groups (**Figures 6A, B**). **Figure 6A** shows the characteristic genera among the Mc, Mm, and Mt groups, with further analysis of the characteristic genera in the Mt group shown in **Figure 6B**. Additionally, random forest analysis was applied to explore nonlinear interdependencies among variables and identify key species that could distinguish differences between groups. **Figure 6C** presents the characteristic genera of the Mt group. We subsequently performed ROC curve analysis on the enriched characteristic genera in the Mt group, using an area under the curve (AUC)>0.8 as the criterion. As shown in **Figure 6D**, the characteristic genera *Lawsonella* (AUC=0.81), *Lutibacter* (AUC=0.85), *Paludibaculum* (AUC=0.90), *Peptostreptococcus* (AUC=0.82), *Staphylococcus* (AUC=0.86), and *Sporosarcina* (AUC=0.92) in the Mt group presented high AUC values, suggesting that these genera may serve as potential biomarkers for Wuling powder in the treatment of IBS-D. By integrating the results of LEfSe analysis, random forest analysis, and ROC curve analysis, we identified objects with high importance scores, AUC>0.8, and significant intergroup differences. We concluded that the characteristic genera *Lutibacter*, *Paludibaculum*, *Peptostreptococcus*, *Staphylococcus*, and *Sporosarcina* in the Mt group represented meaningful differential species among the groups.

#### 3.3.5 Wuling powder changes the function of the intestinal mucosal microbiota in IBS-D mice

**Figure 7:**
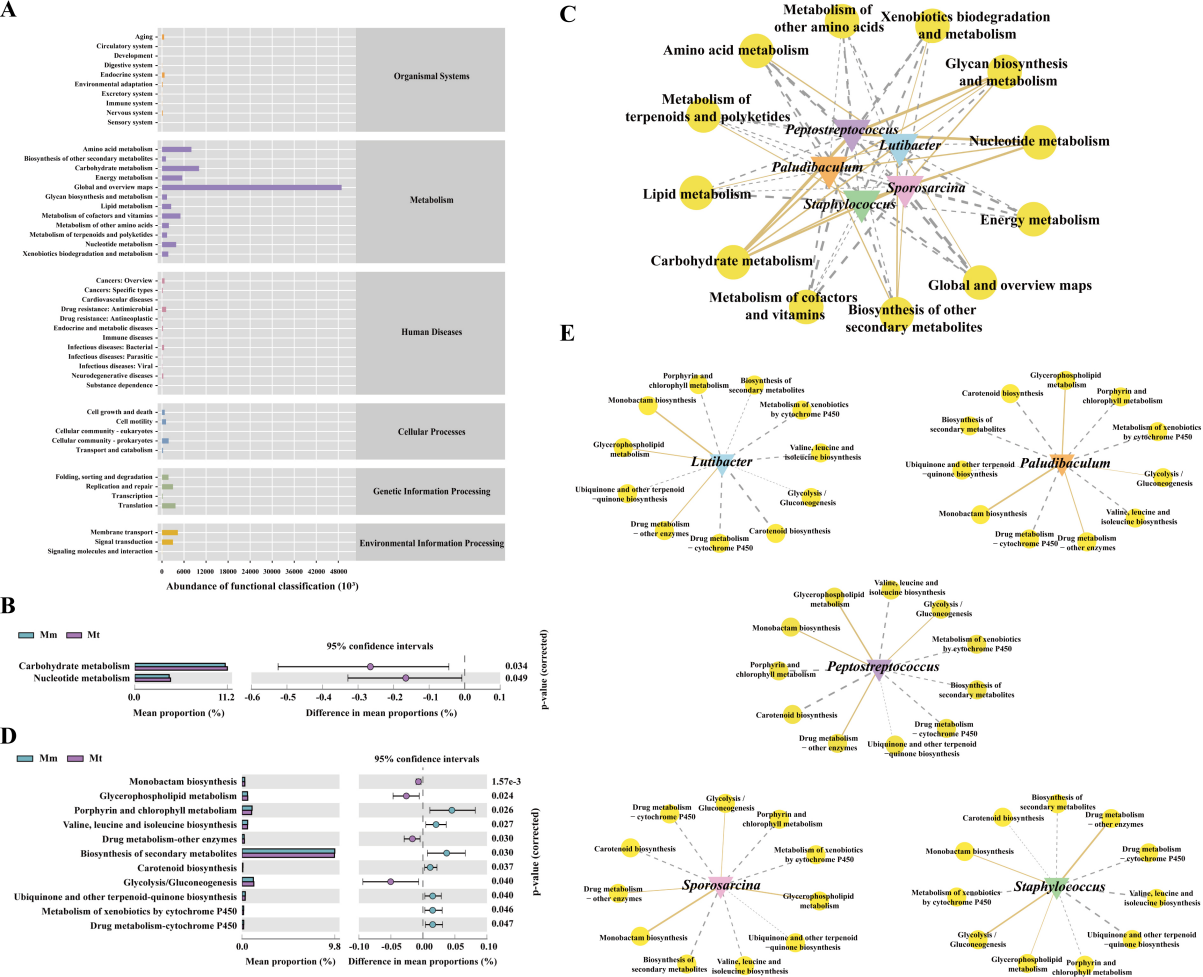
Functional analysis of the intestinal mucosal microbiota in mice. **(A)** Predicted abundance of the KEGG function. The abscissa is the abundance of the metabolic functional classification, the ordinate is the metabolic function at the second level of the classification, and the right-hand side is the first level of classification to which the function belongs. **(B)** Significantly different metabolic functions of the Mt group. **(C)** Interaction network of the “characteristic genus-metabolic function”. **(D)** Significantly different metabolic pathways of the Mt group. **(E)** Interaction of the “characteristic genus–metabolic pathway”. The solid line represents a positive correlation, and the dashed line represents a negative correlation. The thickness of the line indicates the strength of the correlation. Mc, normal control group; Mm, model control group; Mt, treatment group.

To observe whether changes in the intestinal mucosal microbial composition further lead to functional changes, we further predicted microbial function. The gut mucosal microbiota functions were broadly categorized into 1526 primary groups, with 46 subfunctional categories at the second level. Among these, the largest proportion was attributed to the “Metabolism” category, comprising 152 subfunctional groups (**Figure 7A**). As shown in **Figure 7B**, compared with the Mm group, the Mt group presented significant differences in two metabolic functional categories: carbohydrate metabolism and nucleotide metabolism. To explore the correlations between characteristic bacterial genera and metabolic functions during Wuling power intervention in IBS-D patients, we constructed an “interaction network between characteristic bacterial genera and metabolic functions” (**Figure 7C**). The results revealed that *Lutibacter* presented 7 positive and 5 negative correlations with metabolic functions, with the strongest correlation being “carbohydrate metabolism.” *Paludibaculum* and *Sporosarcina* presented 4 positive and 8 negative correlations, with “carbohydrate metabolism” having the strongest correlation for both. *Staphylococcus* and *Peptostreptococcus* presented 3 positive and 9 negative correlations, with the strongest correlations being “carbohydrate metabolism” and “nucleotide metabolism”, respectively. Furthermore, we analyzed the third-level subfunctional categories within the “Metabolism” group. As shown in **Figure 7D**, compared with the Mm group, the Mt group presented significant differences in multiple metabolic pathways, including “Glycolysis/Gluconeogenesis”, “Ubiquinone and other terpenoid-quinone biosynthesis”, “Monobactam biosynthesis”, “Valine, leucine, and isoleucine biosynthesis”, “Glycerophospholipid metabolism”, “Porphyrin and chlorophyll metabolism”, “Carotenoid biosynthesis”, “Metabolism of xenobiotics by cytochrome P450”, “Drug metabolism - cytochrome P450”, “Drug metabolism - other enzymes” and “Biosynthesis of secondary metabolites”. We further conducted a correlation network analysis between the characteristic bacterial genera in the Mt group and the significantly different metabolic pathways. As shown in **Figure 7E**, *Peptostreptococcus* exhibited significant positive correlations with “Glycolysis/Gluconeogenesis”, “Glycerophospholipid metabolism”, “Monobactam biosynthesis”, and “Drug metabolism-other enzymes”, whereas it presented significant negative correlations with “Valine, leucine, and isoleucine biosynthesis”, “Porphyrin and chlorophyll metabolism”, “Carotenoid biosynthesis”, “Metabolism of xenobiotics by cytochrome P450”, “Drug metabolism-cytochrome P450”, and “Biosynthesis of secondary metabolites”. *Paludibaculum* exhibited a significant positive correlation with “monobactam biosynthesis”. *Sporosarcina* was significantly positively correlated with “Glycerophospholipid metabolism” and “Monobactam biosynthesis”, whereas it was significantly negatively correlated with “Porphyrin and chlorophyll metabolism” and “Biosynthesis of secondary metabolites”. Collectively, these findings suggest that the dynamic correlation networks reflect the associations between characteristic microbiota and metabolic functions/pathways, indicating that these metabolic functions and pathways may represent the primary routes through which Wuling power intervention affects the gut mucosal microbiota in IBS-D mice.

#### 3.3.6 Correlation analysis between the characteristic genera, TNF-α, IL-6, and MFGE8

**Figure 8:**
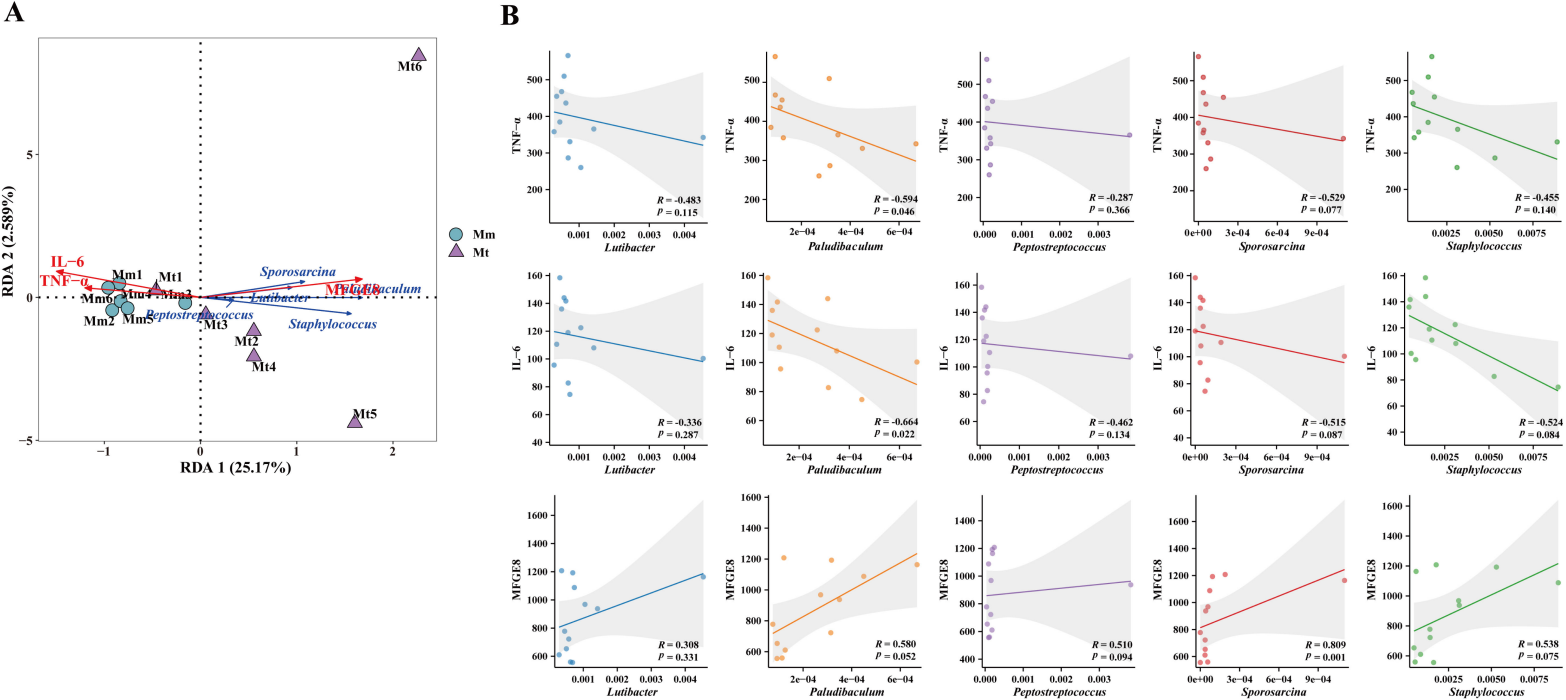
Correlation analysis between the characteristic genera, TNF-α, IL-6, and MFGE8. **(A)** RDA of the Mt group. The angle between the connecting arrows represents the correlation, with an acute angle indicating a positive correlation and an obtuse angle indicating a negative correlation. **(B)** Scatter plot of the correlations between TNF-α, IL-6, and MFGE8 and characteristic genera of the Mt group. Mm, model control group; Mt, treatment group.

To further analyze the effects of Wuling power on the correlation between the intestinal mucosal microbiota and serum inflammation-related indicators in IBS-D mice, we performed RDA and Spearman’s rank correlation coefficient analysis between the characteristic genera TNF-α, IL-6, and MFGE8 in each group. The RDA results revealed that the characteristic genera *Lutibacter*, *Paludibaculum*, *Peptostreptococcus*, *Staphylococcus* and *Sporosarcina* in the Mt group were positively correlated with MFGE8 and negatively correlated with TNF-α and IL-6 (**Figure 8A**). The results of the correlation scatter plot revealed that the characteristic genus *Sporosarcina* in the Mt group was significantly positively correlated with MFGE8 (R=0.809). The level of the *paludibaculum* was significantly negatively correlated with the levels of TNF-α (R=-0.594) and IL-6 (R=-0.664) (**Figure 8B**).

## 4 Discussion

### 4.1 Wuling powder modulates the intestinal mucosal microbiota in IBS-D mice, and this modulation represents a key mechanism underlying its therapeutic efficacy in IBS-D mice

The study of microbial community diversity is highly important for exploring microbial community functions and elucidating the relationships between microbial communities and their habitats (Mitselou et al., 2020). Research shows gut microbiota dysbiosis is common in IBS-D patients, characterized by increased Enterobacteriaceae, decreased *Lactobacillus*, *Bifidobacterium*, and *Prevotella* species (Tao, 2023), and significantly reduced microbial density and diversity (Ji et al., 2016). The significant alterations in gut microbiota composition, notably in the mucosa-associated microbiota, observed in IBS-D patients provide the rationale for selecting small intestinal mucosal samples in this research (Choghakhori et al., 2017). In clinical practice, traditional Chinese medicine and compound prescriptions are usually made into decoctions, which enter the gastrointestinal tract of the human body after oral administration. Compound traditional Chinese medicines can maintain the gut microbiota balance through mechanisms such as modulating microbial proportions, enhancing mucosal barriers, and reducing inflammatory cytokines (Zhang et al., 2021). The therapeutic mechanisms of common prescriptions for IBS-D, such as the Sishen pill, Gegenqinlian decoction, Shenling baizhu powder, and Simo decoction, have been investigated from the perspective of the intestinal microbiota. In contrast, the therapeutic effects of Wuling powder on IBS-D have been explored only through clinical studies. With the widespread application of 16S rRNA high-throughput sequencing technology in microbial community diversity research, we investigated the changes in the intestinal mucosal microbiota structure in IBS-D mice following Wuling powder intervention, providing a microbiota-based therapeutic rationale for the treatment of IBS-D. The sequencing depth was validated via sample dilution curves, Shannon–Wiener curves, and coverage indices, confirming that the sequencing depth was sufficient and reasonable, covering the majority of microbial species and meeting the experimental requirements. Our findings revealed that both the Mm and Mt groups presented alterations in their intestinal mucosal microbial community structure. PCoA analysis demonstrated that both the model group and Wuling powder intervention significantly influenced the structure of the intestinal mucosal microbiota of the mice. Importantly, the microbial communities of the Mt and Mc groups closely aligned and overlapped, indicating that Wuling powder could restore the microbial community structure.

The gut microbiota, a vast and diverse microbial community primarily composed of Firmicutes, Bacteroidetes, Proteobacteria, and Actinobacteria, plays crucial roles in maintaining intestinal homeostasis. Among these, Firmicutes and Bacteroidetes predominate, collectively accounting for over 90% of the total microbial population (Shrestha et al., 2022). Our study demonstrated that Wuling power altered the relative abundances of major bacterial phyla and genera in the small intestine of model mice. At the phylum level, the Mm group exhibited decreased relative abundances of beneficial bacteria (Firmicutes and Bacteroidota) and increased relative abundances of potentially harmful bacteria (Proteobacteria and Actinobacteria) compared to the Mc group. At the genus level, beneficial genera *Streptococcus* and *Candidatus_Arthromitus* were reduced in the Mm group. Following Wuling power intervention, these alterations were ameliorated, with beneficial bacteria upregulated and harmful bacteria downregulated, resulting in a profile tending toward that of the Mc group. We subsequently analyzed the top 10 dominant phyla and genera with relative abundances greater than 1%. The results showed that Firmicutes, Bacteroidetes, Proteobacteria, Actinobacteria, *Streptococcus*, and *Candidatus-Arthromitus* may play essential roles in IBS-D treatment with Wuling powder. *Streptococcus* represents a heterogeneous group of gram-positive bacteria with significant implications in both medical and industrial contexts. These organisms constitute integral components of the normal microbiota in animals and humans and play crucial ecological roles. Notably, certain species within this genus are capable of inducing a spectrum of diseases ranging from subacute to acute and even chronic conditions (Bai et al., 2022). The *Candidatus-Arthromitu* strain segmented filamentous bacteria-mouse-NL is a commensal bacterium necessary for inducing the postnatal maturation of homeostatic innate and adaptive immune responses in the mouse gut. It closely combines with the surface of the absorptive intestinal epithelium without causing an inflammatory reaction (Bolotin et al., 2014), which is consistent with our findings. LEfSe analysis, random forest analysis, and ROC curve analysis revealed that the characteristic genera *Lutibacter*, *Paludibaculum*, *Peptostreptococcus*, *Staphylococcus*, and *Sporosarcina* were significantly enriched in the Mt group, suggesting that the above genera could be used as essential biomarkers in IBS-D treatment with Wuling powder. *Lutibacter* is a genus of Bacteroidota. At present, only some species of *Lutibacter* have been isolated from marine sediments, and no research on intestinal diseases has been reported (Du et al., 2020). *Paludibaculum* is a genus of Acidobacteriota, and there are few reports about this genus. *Paludibaculum fermentans* has been reported as a facultatively anaerobic, heterotrophic acidobacterium capable of dissimilatory Fe (III) reduction in coastal wetland environments (Dedysh et al., 2021). Further research suggests (Aidah et al., 2023; Yin et al., 2023) *Paludibaculum* may contribute to maternal-fetal microbial translocation hematogenously, though its direct relationship with gut microbiota and intestinal inflammation requires further study. Future work should examine its gut colonization patterns and potential role in inflammatory diseases. The genera Firmicutes include *Peptostreptococcus*, *Staphylococcus*, and *Sporosarcina*. Previous research has established that (Wlodarska et al., 2017) the symbiotic bacterium *Peptostreptococcus* russellii enhances intestinal barrier function and suppresses excessive immune responses through mucin utilization and tryptophan metabolism, generating metabolites (such as IA) with anti-inflammatory and antioxidant properties. Its functional impairment in diseases like IBD may contribute to dysbiosis and exacerbated inflammation. Consequently, this bacterium or its metabolites represent potential probiotics or therapeutic targets. Recent studies demonstrate that (Liu et al., 2024; Shen et al., 2024) *Peptostreptococcus* anaerobius and P. stomatis drive intestinal inflammation and tumorigenesis by disrupting microbiota homeostasis and activating pro-inflammatory pathways, including TLR/NLRP3 and ERBB2-MAPK. *Staphylococcus*, a ubiquitous Gram-positive genus including species such as S. aureus and S. epidermidis, is increasingly linked by evidence to the initiation and progression of various cancers (Wei et al., 2022). However, *Sporosarcina* has not been reported to be related to intestinal diseases. Furthermore, we conducted a correlation network analysis between these characteristic genera and metabolic functions, revealing that the characteristic genera in the Mt group were significantly positively correlated with carbohydrate metabolism and nucleotide metabolism. Additionally, in the analysis of the correlation network between characteristic genera and metabolic pathways, we found that monobactam biosynthesis was significantly positively correlated with *Peptostreptococcus*, *Paludibaculum*, and *Sporosarcina*. Monobactam biosynthesis is classified under the secondary category of “Biosynthesis of other secondary metabolites” in the KEGG metabolic functional classification. Studies have demonstrated that during specific growth phases of microorganisms (typically during the late growth stage), secondary metabolites are synthesized using primary metabolites as precursors (Dinglasan et al., 2025). These compounds often lack clearly defined functional roles in life processes, exhibit complex molecular structures, and can be categorized into various groups, such as pigments, antibiotics, hormones, alkaloids, and toxins. Furthermore, the gut microbiota is closely associated with human health and disease, with recent domestic and international research revealing the intimate relationship between the gut microbiota and host diseases. This highlights the crucial role of the gut microbiota and its bioactive secondary metabolites in maintaining host homeostasis (Anwer et al., 2025).

In summary, we speculate that Wuling powder may exert its therapeutic effect on IBS-D mice by altering the composition and function of the colonic mucosal microbiota, thereby facilitating the biosynthesis of other secondary metabolites. Utilizing 16S rRNA sequencing, this study first revealed the potential mechanism of Wuling power for treating IBS-D via gut microbiota. It preliminarily identified characteristic biomarkers in the intestinal mucosal microbiota and predicted related metabolic pathways. However, methodological limitations preclude validation through functional studies like metabolomics or microbial culture. Subsequent research must integrate multidisciplinary approaches to fully elucidate Wuling power’s mechanism of action and its associated metabolic regulatory networks. Furthermore, to elucidate the critical role of the gut microbiota as an intermediary in drug efficacy transmission, we recommend the incorporation of fecal microbiota transplantation (FMT) experiments in future studies. Specifically, standardized suspensions of microbiota from Mt group mice could be transplanted into untreated IBS-D Mm mice to validate the causal relationship between microbiota remodeling and symptom alleviation. Notably, while this study has provided preliminary evidence linking microbial community shifts to the pharmacological effects of this compound, FMT validation experiments are pending owing to experimental limitations, which we prioritize in subsequent research efforts.

### 4.2 Wuling power significantly reduced serum IL-6 and TNF-α levels, increased MFGE8 levels, and highlighted the correlation between gut mucosal bacterial genera and environmental factors, emphasizing its role in mediating its therapeutic effects in IBS-D mice

Studies have demonstrated that inflammatory responses play a pivotal role in the pathogenesis of IBS-D (Ji and Zhang, 2022). Inflammation, as a complex biological process, represents the body’s defense mechanism against tissue injury or infection. The activation of inflammatory responses is closely associated with the production of various proinflammatory cytokines, among which TNF-α and IL-6 are identified as the primary proinflammatory mediators in this process. Elevated levels of pro-inflammatory cytokines are closely associated with IBS-D. Clinical studies have demonstrated that patients with IBS-D exhibit elevated levels of proinflammatory cytokines, such as TNF-αand IL-6, which play a significant role in the pathogenesis of IBS-D (Ji et al., 2016; Mitselou et al., 2020). Animal studies demonstrate that (Chen et al., 2023) elevated TNF-α and IL-6 levels mediate low-grade intestinal inflammation, compromise the gut mucosal barrier, and sensitize visceral sensory nerves, collectively driving the core symptoms of IBS-D, including diarrhea and abdominal pain. MFGE8, initially identified on the surface of milk fat globules, is a transmembrane glycoprotein involved in various processes, including inflammation, immunity, apoptosis, proliferation, and tumor progression. Notably, MFGE8 has been shown to effectively alleviate intestinal inflammation, reduce the production of proinflammatory cytokines such as TNF-α and IL-6, and enhance the integrity of the intestinal mucosal barrier (Zhang et al., 2021). Intestinal fibrosis, recognized as a characteristic complication of chronic gut inflammation, is shown in Crohn’s disease (CD) studies to be prevented and reversed by MFGE8 in experimental models (Lin et al., 2024). These findings establish MFGE8 as a key contributor to intestinal fibrogenesis and a potential therapeutic target. The results revealed that the levels of TNF-α and IL-6 in the serum of the mice in the Mm group were extremely significantly increased compared with those in the Mc group, whereas the MFGE8 level was extremely significantly decreased. Compared with those in the Mm group, the levels of TNF-αand IL-6 in the Mt group were significantly lower, and the MFGE8 level was extremely significantly greater. Our findings suggest Wuling power suppresses intestinal inflammation in IBS-D mice, likely by downregulating TNF-α and IL-6 while upregulating MFGE8. Elevated MFGE8 implies enhanced mucosal repair and attenuated immune responses, functioning as a key bridging molecule facilitating apoptotic cell clearance and inflammation resolution. In atherosclerosis models (Zhang et al., 2021), MFGE8 deficiency exacerbates inflammation by impairing phagocytic clearance and activating MAPK/NF-κB signaling. Its restoration attenuates TNF-α/IL-6production. Future studies should examine whether MFGE8 serves as a core regulator bridging IBS-D-associated gut microbiota to inflammation resolution, to elucidate its anti-inflammatory. This study identified significant correlations between host-derived factors (TNF-α, IL-6, MFGE8) and gut mucosal microbiota. In the Mt group, *Sporosarcina* positively correlated with MFGE8, whereas *Paludibaculum* negatively correlated with TNF-α and IL-6. These findings suggest microbiota influence TNF-α, IL-6, and MFGE8 levels. *Paludibaculum* belongs to the Bacteria phylum, and *Sporosarcina* belongs to the Firmicutes phylum. Reports associating *Paludibaculum* with intestinal diseases are presented in Section 4.1 (Discussion), whereas no reports have been found linking *Sporosarcina* to intestinal disorders. In summary, there was a significant negative regulatory effect between the intestinal mucosal characteristic genera and the inflammatory response in the IBS-D treatment with Wuling powder.

In summary, our findings demonstrate that Wuling powder intervention effectively reduces the expression of the inflammatory cytokines TNF-α and IL-6 while increasing MFGE8 levels, thereby improving dysbiosis associated with IBS-D and its systemic complications. While our findings underscore the regulatory effects of Wuling powder, the molecular mechanisms underlying its action remain incompletely understood. Further investigation into the molecular mechanisms by which Wuling powder modulates the gut mucosal microbiota and host metabolism in IBS-D mice is necessary for optimizing its therapeutic applications.

## 5 Conclusion

**Figure 9:**
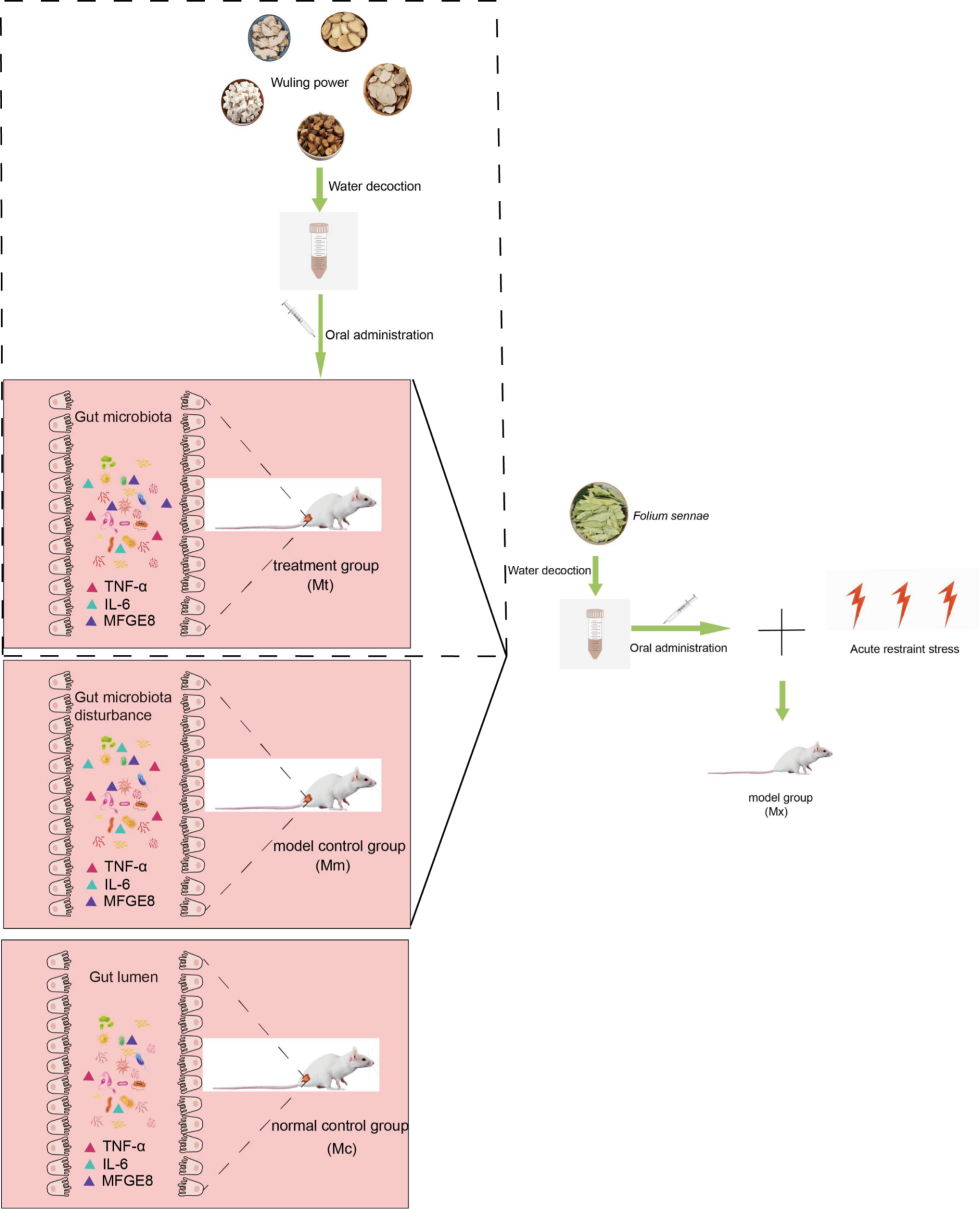
Regulatory effects of Wuling power decoction on gut mucosal microbiota and serum inflammatory factors. Oral administration of Wuling power decoction to IBS-D model mice induced by *Folium sennae* combined with acute restraint stress resulted in significant alterations in gut microbiota structure and serum inflammatory factors (TNF-α, IL-6, MFGE8). Compared to the Mm group, the Mt group exhibited improved microbiota composition, significantly reduced pro-inflammatory cytokines, and elevated levels of the barrier-repairing factor MFGE8. The effects observed in the Mt group approached those seen in the Mc group. This figure was created using Adobe Illustrator 2025 software. Mc, normal control group; Mm, model control group; Mt, treatment group.

Wuling powder can improve the inflammatory response, intestinal mucosal microbiota structure, and function in IBS-D mice. The experimental results suggest that Wuling powder may inhibit the occurrence of the inflammatory response by decreasing the TNF-α and IL-6 levels and increasing the MFGE8 level and may achieve the effect of IBS-D treatment by regulating the intestinal mucosal microbiota structure and function, which provides a new idea for the clinical prevention and treatment of IBS-D via Wuling powder (**Figure 9**). However, the intestinal mucosal microbiota and metabolic pathways involved in the pathogenesis of IBS-D are complex and have not yet been fully detected and elucidated. Therefore, the specific mechanism of the intestinal mucosal microbiota needs further research and verification.

## Data Availability

All authors are requested to make sure that all data and materials as well as software application or custom code support their published claims and comply with field standards. The intestinal mucosal microbiota sequencing data has been uploaded to the NCBI database (https://www.ncbi.nlm.nih.gov/), no. PRJNA951492.

## References

[B1] Aidah N. Engeu O. P. Baptist T. J. Muwanika V. B. Johnson M. Joel B. (2023). Diversity of bacterial community in the rhizosphere and bulk soil of Artemisia annua grown in highlands of Uganda. PloS One 18, e0269662. doi: 10.1371/journal.pone.0269662, PMID: 36952433 PMC10035820

[B2] Anwer E. K. E. Ajagbe M. Sherif M. Musaibah A. S. Mahmoud S. ElBanbi A.. (2025). Gut microbiota secondary metabolites: key roles in GI tract cancers and infectious diseases. Biomedicines 13, 100. doi: 10.3390/biomedicines13010100, PMID: 39857684 PMC11762448

[B3] Bai H. Mu L. Qiu L. Chen N. Li J. Zeng Q.. (2022). Complement C3 regulates inflammatory response and monocyte/macrophage phagocytosis of streptococcus agalactiae in a teleost fish. Int. J. Mol. Sci. 23, 15586. doi: 10.3390/ijms232415586, PMID: 36555227 PMC9779060

[B4] Bellini M. Gambaccini D. Stasi C. Urbano M. T. Marchi S. Usai-Satta P. (2014). Irritable bowel syndrome: a disease still searching for pathogenesis, diagnosis and therapy. World J. Gastroenterol. 20, 8807–8820. doi: 10.3748/wjg.v20.i27.8807, PMID: 25083055 PMC4112881

[B5] Bolotin A. de Wouters T. Schnupf P. Bouchier C. Loux V. Rhimi M.. (2014). Genome sequence of “Candidatus arthromitus” sp. Strain SFB-mouse-NL, a commensal bacterium with a key role in postnatal maturation of gut immune functions. Genome announcements 2, e00705–e00714. doi: 10.1128/genomeA.00705-14, PMID: 25035333 PMC4102870

[B6] Canakis A. Haroon M. Weber H. C. (2020). Irritable bowel syndrome and gut microbiota. Curr. Opin. endocrinol. diabetes Obes. 27, 28–35. doi: 10.1097/MED.0000000000000523, PMID: 31789724

[B7] Chen J. Lan H. Li C. Xie Y. Cheng X. Xia R.. (2024). Gut microbial signatures of patients with diarrhea-predominant irritable bowel syndrome and their healthy relatives. J. Appl. Microbiol. 135, lxae118. doi: 10.1093/jambio/lxae118, PMID: 38849305

[B8] Chen Q. Zhang H. Sun C. Y. He Q. Y. Zhang R. R. Luo B. F.. (2023). Evaluation of two laboratory model methods for diarrheal irritable bowel syndrome. Mol. Med. (Cambridge Mass.) 29, 5. doi: 10.1186/s10020-022-00599-x, PMID: 36635623 PMC9837933

[B9] Choghakhori R. Abbasnezhad A. Hasanvand A. Amani R. (2017). Inflammatory cytokines and oxidative stress biomarkers in irritable bowel syndrome: Association with digestive symptoms and quality of life. Cytokine 93, 34–43. doi: 10.1016/j.cyto.2017.05.005, PMID: 28506572

[B10] Dedysh S. N. Beletsky A. V. Kulichevskaya I. S. Mardanov A. V. Ravin N. V. (2021). Complete genome sequence of paludibaculum fermentans P105T, a facultatively anaerobic acidobacterium capable of dissimilatory fe (III) reduction. Microbiol. resource announcements 10, e01313–e01320. doi: 10.1128/MRA.01313-20, PMID: 33414319 PMC8407742

[B11] Di J. Xie S. Shen J. Fang L. Tan Z. Liang X. (2025). Arecoline triggers psychostimulant responses by modulating the intestinal microbiota to influence neurotransmitter levels and digestive enzyme activity. Pharm. (Basel Switzerland) 18, 794. doi: 10.3390/ph18060794, PMID: 40573191 PMC12196527

[B12] Ding X. Li S. Huang H. Shen J. Ding Y. Chen T.. (2024). Bioactive triterpenoid compounds of Poria cocos (Schw.) Wolf in the treatment of diabetic ulcers via regulating the PI3K-AKT signaling pathway. J. ethnopharmacol. 325, 117812. doi: 10.1016/j.jep.2024.117812, PMID: 38301984

[B13] Dinglasan J. L. N. Otani H. Doering D. T. Udwary D. Mouncey N. J. (2025). Microbial secondary metabolites: advancements to accelerate discovery towards application. Nat. Rev. Microbiol. 23, 338–354. doi: 10.1038/s41579-024-01141-y, PMID: 39824928

[B14] Du Z. Z. Zhou L. Y. Wang T. J. Li H. R. Du Z. J. (2020). Lutibacter citreus sp. nov., isolated from Arctic surface sediment. Int. J. systematic evolutionary Microbiol. 70, 3154–3161. doi: 10.1099/ijsem.0.004146, PMID: 32302274

[B15] Fang L. Shen J. Wu Y. Tan Z. (2025). Involvement of intestinal mucosal microbiota in adenine-induced liver function injury. 3 Biotech. 15, 6. doi: 10.1007/s13205-024-04180-7, PMID: 39676888 PMC11638458

[B16] Galica A. N. Galica R. Dumitraşcu D. L. (2022). Diet, fibers, and probiotics for irritable bowel syndrome. J. Med. Life. 15, 174–179. doi: 10.25122/jml-2022-0028, PMID: 35419092 PMC8999090

[B17] Gao S. J. (2019). Observation of the curative effect of wuling decoctiong in the treatment of diarrhea irritable bowel syndrome of spleen deficiency and dampness obstruction. Fujian University of Traditional Chinese Medicine, Fuzhou, Fujian Province, China.

[B18] Grayson M. (2016). Irritable bowel syndrome. Nature 533, S101. doi: 10.1038/533S101a, PMID: 27191484

[B19] Guo M. Di J. Tan Z. Xiao N. Peng M. (2025). One of the short-chain fatty acids (SCFAs), sodium propionate, can reduce the dosage of sishen pill in regulating the intestinal microbiota in diarrhea with kidney-yang deficiency syndrome. J. Inflammation Res. 18, 7195–7214. doi: 10.2147/JIR.S522689, PMID: 40491784 PMC12147927

[B20] Hong G. Li Y. Yang M. Li G. Qian W. Xiong H.. (2020). Gut fungal dysbiosis and altered bacterial-fungal interaction in patients with diarrhea-predominant irritable bowel syndrome: An explorative study. Neurogastroenterol. motility. 32, e13891. doi: 10.1111/nmo.13891, PMID: 32449259

[B21] Hu J. Peng M. J. Luo H. H. Ou Y. N. Wu Y. G. Xiao N. Q. (2018). Effects of Senna on the intestinal microbiota and enzyme activity in mice with spleen-deficiency. Chin. J. Microecol. 30, 155–157. doi: 10.13381/j.cnki.cjm.201802007

[B22] Hua X. Li B. Yu F. Zhao W. Tan Y. Li X.. (2022). Protective effect of MFG-E8 on necroptosis-induced intestinal inflammation and enteroendocrine cell function in diabetes. Nutrients 14, 604. doi: 10.3390/nu14030604, PMID: 35276962 PMC8839169

[B23] Ji M. Huang H. Lan X. M. Zhang A. H. (2016). Changes of intestinal microflora in different irritable bowel syndrome subtypes. J. Clin. Gastroenterol. 28, 103–106. doi: 10.3870/1cxh.j.issn.1005-541X.2016.02.12

[B24] Ji S. Zhang Q. (2022). Momordica charantia polysaccharides alleviate diarrhea-predominant irritable bowel syndrome by regulating intestinal inflammation and barrier via NF-κB pathway. Allergologia immunopathologia 50, 62–70. doi: 10.15586/aei.v50i3.584, PMID: 35527657

[B25] Jun H. Ko S. J. Kim K. Kim J. Park J. W. (2022). An overview of systematic reviews of herbal medicine for irritable bowel syndrome. Front. Pharmacol. 13. doi: 10.3389/fphar.2022.894122, PMID: 35662700 PMC9158123

[B26] Kasti A. Katsas K. Nikolaki M. D. Triantafyllou K. (2025). The role and the regulation of NLRP3 inflammasome in irritable bowel syndrome: A narrative review. Microorganisms 13, 171. doi: 10.3390/microorganisms13010171, PMID: 39858939 PMC11767632

[B27] Li C. Zhou K. Xiao N. Peng M. Tan Z. (2022). The effect of qiweibaizhu powder crude polysaccharide on antibiotic-associated diarrhea mice is associated with restoring intestinal mucosal bacteria. Front. Nutr. 9. doi: 10.3389/fnut.2022.952647, PMID: 35873450 PMC9305308

[B28] Li L. Long Q. Deng N. Tan Z. (2025). Association of intestinal mucosal barrier function with intestinal microbiota in Spleen-Kidney Yang Deficiency IBS-D mice. Front. Microbiol. 16. doi: 10.3389/fmicb.2025.1567971, PMID: 40365066 PMC12069268

[B29] Lin X. X. Guo S. J. (2020). Therapeutic efficacy of modified wuling power plus individualized diet in diarrhea-predominant irritable bowel syndrome (IBS-D): an analysis. Chin. J. Integr. Tradit. West. Med. Dig. 28, 891–894. doi: 10.3969/j.issn.1671-038X.2020.11.16

[B30] Lin S. Wang J. Mukherjee P. K. Mao R. West G. Czarnecki D.. (2024). Milk fat globule-epidermal growth factor 8 (MFGE8) prevents intestinal fibrosis. Gut 73, 1110–1123. doi: 10.1136/gutjnl-2022-328608, PMID: 38378253 PMC11248270

[B31] Liu B. N. Liu X. T. Liang Z. H. Wang J. H. (2021). Gut microbiota in obesity. World J. gastroenterol. 27, 3837–3850. doi: 10.3748/wjg.v27.i25.3837, PMID: 34321848 PMC8291023

[B32] Liu Y. Wong C. C. Ding Y. Gao M. Wen J. Lau H. C.. (2024). Peptostreptococcus anaerobius mediates anti-PD1 therapy resistance and exacerbates colorectal cancer via myeloid-derived suppressor cells in mice. Nat. Microbiol. 9, 1467–1482. doi: 10.1038/s41564-024-01695-w, PMID: 38750176 PMC11153135

[B33] Liu Wu Y. Tan Z. J. (2020). Establishment of a mouse model of Ganqichengpi diarrhea and the efficacy of Tongxieyaofang prescription. Chin. J. Appl. Environ. Biol. 26, 1023–1027. doi: 10.19675/j.cnki.1006-687x.2019.09026

[B34] Mearin F. Lacy B. E. Chang L. Chey W. D. Lembo A. J. Simren M.. (2016). Bowel disorders. Gastroenterology S0016-5085(16)00222-5. doi: 10.1053/j.gastro.2016.02.031, PMID: 27144627

[B35] Mitselou A. Grammeniatis V. Varouktsi A. Papadatos S. S. Katsanos K. Galani V. (2020). Proinflammatory cytokines in irritable bowel syndrome: a comparison with inflammatory bowel disease. Intestinal Res. 18, 115–120. doi: 10.5217/ir.2019.00125, PMID: 32013318 PMC7000645

[B36] Peng Y. Xu L. H. (2021). Establishment and evaluation of mouse model of diarrhea-predominant irritable bowel syndrome. Acta Univ. Med. Anhui. 56, 1152–1155. doi: 10.19405/j.cnki.issn1000-1492.2021.07.028

[B37] Qu Y. Park S. H. Dallas D. C. (2023). The role of bovine kappa-casein glycomacropeptide in modulating the microbiome and inflammatory responses of irritable bowel syndrome. Nutrients 15, 3991. doi: 10.3390/nu15183991, PMID: 37764775 PMC10538225

[B38] Quaglio A. E. V. Grillo T. G. De Oliveira E. C. S. Di Stasi L. C. Sassaki L. Y. (2022). Gut microbiota, inflammatory bowel disease and colorectal cancer. World J. gastroenterol. 28, 4053–4060. doi: 10.3748/wjg.v28.i30.4053, PMID: 36157114 PMC9403435

[B39] Saha L. (2014). Irritable bowel syndrome: pathogenesis, diagnosis, treatment, and evidence-based medicine. World J. gastroenterol. 20, 6759–6773. doi: 10.3748/wjg.v20.i22.6759, PMID: 24944467 PMC4051916

[B40] Shen J. Fang L. Tan Z. Xiao N. Peng M. (2025a). The effects of functional biscuits on intestinal mucosal microbiota composition, brain function, and antioxidant activity. Biosci. microbiota Food Health 44, 171–181. doi: 10.12938/bmfh.2024-078, PMID: 40171390 PMC11957763

[B41] Shen J. Fang L. Wu Y. Deng N. Peng X. Li D.. (2025b). Intestinal microbiota dysbiosis disrupts the mucosal barrier, triggering inflammatory responses in gut-kidney interaction and exacerbating diarrhea. J. Inflammation Res. 18, 9379—9399. doi: 10.2147/JIR.S522689, PMID: 40687146 PMC12276751

[B42] Shen X. H. Guan J. Lu D. P. Hong S. C. Yu L. Chen X. (2024). Peptostreptococcus Anaerobius enhances dextran sulfate sodium-induced colitis by promoting nf-κB-NLRP3-Dependent macrophage pyroptosis. Virulence 15, 2435391. doi: 10.1080/21505594.2024.2435391, PMID: 39611567 PMC11610558

[B43] Shen J. Zhou M. Xiao N. Tan Z. Liang X. (2025c). Unveiling the mystery of the stimulatory effects of arecoline: its relevance to the regulation of neurotransmitters and the microecosystem in multi-ecological intestinal sites. Int. J. Mol. Sci. 26, 3150. doi: 10.3390/ijms26073150, PMID: 40243919 PMC11989758

[B44] Shrestha B. Patel D. Shah H. Hanna K. S. Kaur H. Alazzeh M. S.. (2022). The role of gut-microbiota in the pathophysiology and therapy of irritable bowel syndrome: A systematic review. Cureus 14, e28064. doi: 10.7759/cureus.28064, PMID: 36127988 PMC9477602

[B45] Tanaka Y. Yamashita R. Kawashima J. Mori H. Kurokawa K. Fukuda S.. (2022). Omics profiles of fecal and oral microbiota change in irritable bowel syndrome patients with diarrhea and symptom exacerbation. J. gastroenterol. 57, 748–760. doi: 10.1007/s00535-022-01888-2, PMID: 35908139 PMC9522833

[B46] Tao Y. (2023). Analysis of the gut microbiological characteristics of patients with diarrheal irritable bowel syndrome and its association with somatic and psychiatric symptoms. Chongqing Medical University, Chongqing Municipality, China.

[B47] Tao L. Xu H. He Q. (2022). Potential influences of expression levels of MFGE8 and HMGB1 on the intestinal mucosal barrier function and inflammatory response after blunt abdominal injury in rats. Acta cirurgica brasileira 37, e370303. doi: 10.1590/acb370303, PMID: 35674581 PMC9161623

[B48] Wang S. Jing W. Gu G. Li S. Pang J. Cong H.. (2025). Improvement effect and mechanism of XuanFuDaiZhe tang on rats with diarrheal irritable bowel syndrome induced by colorectal dilation. J. ethnopharmacol. 337, 118938. doi: 10.1016/j.jep.2024.118938, PMID: 39419305

[B49] Wei Y. Sandhu E. Yang X. Yang J. Ren Y. Gao X. (2022). Bidirectional functional effects of staphylococcus on carcinogenesis. Microorganisms 10, 2353. doi: 10.3390/microorganisms10122353, PMID: 36557606 PMC9783839

[B50] Wlodarska M. Luo C. Kolde R. d’Hennezel E. Annand J. W. Heim C. E.. (2017). Indoleacrylic acid produced by commensal peptostreptococcus species suppresses inflammation. Cell Host Microbe 22, 25–37.e6. doi: 10.1016/j.chom.2017.06.007, PMID: 28704649 PMC5672633

[B51] Wu Y. Peng X. Li X. Li D. Tan Z. Yu R. (2022). Sex hormones influence the intestinal microbiota composition in mice. Front. Microbiol. 13. doi: 10.3389/fmicb.2022.964847, PMID: 36386696 PMC9659915

[B52] Wu H. Zhan K. Rao K. Zheng H. Qin S. Tang X.. (2022). Comparison of five diarrhea-predominant irritable bowel syndrome (IBS-D) rat models in the brain-gut-microbiota axis. Biomed. pharmacother. = Biomed. pharmacotherapie 149, 112811. doi: 10.1016/j.biopha.2022.112811, PMID: 35303570

[B53] Xu S. S. (2021). Clinical observation of Tongxie Yaofang and Wuling Powder in the treatment of IBS-D with stagnation of liver and deficiency of spleen. Guangzhou University of Chinese Medicine, Guangzhou, Guangdong Province, China. doi: 10.27044/d.cnki.ggzzu.2021.000439

[B54] Yi X. Zhou K. Deng N. Cai Y. Peng X. Tan Z. (2023). Simo decoction curing spleen deficiency constipation was associated with brain-bacteria-gut axis by intestinal mucosal microbiota. Front. Microbiol. 14. doi: 10.3389/fmicb.2023.1090302, PMID: 36846756 PMC9947565

[B55] Yin J. J. Qin S. Wu H. M. Zheng H. Rao K. H. Huang S. G. (2022). Study on constitution distribution characteristics of irritable bowel syndrome based on data mining. New J. Tradit. Chin. Med. 54, 20–24. doi: 10.13457/j.cnki.jncm.2022.11.004

[B56] Yin H. Yu J. Wu W. Li X. Hu R. (2023). Analysis of the microbiome in maternal, intrauterine and fetal environments based on 16S rRNA genes following different durations of membrane rupture. Sci. Rep. 13, 15010. doi: 10.1038/s41598-023-41777-z, PMID: 37696898 PMC10495440

[B57] Yu D. Xie S. Guo M. Wu Y. Tian Q. Wang Z.. (2024). External damp environment aggravates diarrhea in spleen deficiency and dampness syndrome in mice: involvement of small intestinal contents microbiota, energy metabolism, gastrointestinal and fluid functions. Front. Cell. infection Microbiol. 14. doi: 10.3389/fcimb.2024.1495311, PMID: 39544280 PMC11560853

[B58] Yuan Y. Wang X. Huang S. Wang H. Shen G. (2023). Low-level inflammation, immunity, and brain-gut axis in IBS: unraveling the complex relationships. Gut Microbes 15, 2263209. doi: 10.1080/19490976.2023.2263209, PMID: 37786296 PMC10549202

[B59] Zhang Y. Ding J. Wang Y. Feng X. Du M. Liu P. (2021). Guanxinkang decoction attenuates the inflammation in atherosclerosis by regulating efferocytosis and MAPKs signaling pathway in LDLR-/- mice and RAW264.7 cells. Front. Pharmacol. 12. doi: 10.3389/fphar.2021.731769, PMID: 34950025 PMC8688952

[B60] Zhang Q. W. Yang M. J. Liao C. Y. Taha R. Li Q. Y. Abdelmotalab M. I.. (2025). Atractylodes macrocephala Koidz polysaccharide ameliorates DSS-induced colitis in mice by regulating the gut microbiota and tryptophan metabolism. Br. J. Pharmacol. 182, 1508–1527. doi: 10.1111/bph.17409, PMID: 39667762

[B61] Zhao Y. Zhan J. Sun C. Zhu S. Zhai Y. Dai Y.. (2024). Sishen Wan enhances intestinal barrier function via regulating endoplasmic reticulum stress to improve mice with diarrheal irritable bowel syndrome. Phytomed.: Int. J. phytother. phytopharmacol. 129, 155541. doi: 10.1016/j.phymed.2024.155541, PMID: 38579640

